# Patterns of thermal adaptation in a globally distributed plant pathogen: Local diversity and plasticity reveal two‐tier dynamics

**DOI:** 10.1002/ece3.8515

**Published:** 2022-01-26

**Authors:** Anne‐Lise Boixel, Michaël Chelle, Frédéric Suffert

**Affiliations:** ^1^ Université Paris‐Saclay, INRAE, UR BIOGER Thiverval‐Grignon France; ^2^ Université Paris‐Saclay, INRAE, AgroParisTech, UMR ECOSYS Thiverval‐Grignon France

**Keywords:** ecological patterns, environmental heterogeneity, functional diversity, interindividual variation, plasticity, reaction norm, seasonal changes, *Zymoseptoria tritici*

## Abstract

Plant pathogen populations inhabit patchy environments with contrasting, variable thermal conditions. We investigated the diversity of thermal responses in populations sampled over contrasting spatiotemporal scales, to improve our understanding of their dynamics of adaptation to local conditions. Samples of natural populations of the wheat pathogen *Zymoseptoria tritici* were collected from sites within the Euro‐Mediterranean region subject to a broad range of climatic conditions. We tested for local adaptation, by accounting for the diversity of responses at the individual and population levels on the basis of key thermal performance curve parameters and “thermotype” (groups of individuals with similar thermal responses) composition. The characterization of phenotypic responses and genotypic structure revealed the following: (i) a high degree of individual plasticity and variation in sensitivity to temperature conditions across spatiotemporal scales and populations; and (ii) geographic variation in thermal response among populations, with major alterations due to seasonal patterns over the wheat‐growing season. The seasonal shifts in functional composition suggest that populations are locally structured by selection, contributing to adaptation patterns. Further studies combining selection experiments and modeling are required to determine how functional group selection drives population dynamics and adaptive potential in response to thermal heterogeneity.

## Graphical abstract

We investigated the diversity of thermal responses in fungal plant populations sampled within the Euro‐Mediterranean region. We highlighted (i) a high degree of individual plasticity and variation in sensitivity to temperature conditions across spatiotemporal scales and populations; and (ii) geographic variation in thermal response among populations, with major alterations due to seasonal patterns. The seasonal shifts in functional composition suggest that populations are locally structured by selection and thus reveal two‐tier adaptive dynamics.

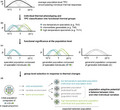

## 1 INTRODUCTION

Environmental heterogeneity (Li & Reynolds, 1995) is regarded as one of the most important elements driving the emergence and maintenance of genetic variation within populations (Hedrick, 1986; Hughes et al., 2008; Levins, 1974; Ravigné et al., 2009) as it dictates physiological responses (Cavieres & Sabat, 2008) and can drive the emergence of local adaptation patterns (Nuismer & Gandon, 2008; Thompson, 2005). Gathering information about the way a given community, species, or population copes with this environmental heterogeneity is crucial for the understanding and prediction of its distribution and responses to current and future environmental changes (Austin, 2007).

The adequate capture of eco‐evolutionary responses requires an integration of physiological variation across biological (individual, group, population, species) and spatiotemporal (seasonal, geographic) scales, given the significant implications of this variation for dynamics (Saloniemi, 1993; Schreiber et al., 2011; Vindenes et al., 2008). It is therefore important to go beyond summarizing diversity through average trait values (Bolnick et al., 2011; Violle et al., 2012), and to account for the individual specialization of phenotypic responses by taking into account both phenotypic plasticity (within‐individual differences; Pigliucci, 2001) and interindividual variation (between‐individual differences; Dall et al., 2012).

The ecological concept of “reaction norm,” describing the set of phenotypes generated by a given genotype in different environments (Schlichting & Pigliucci, 1998), is particularly effective as a tool for accounting for individual specialization (Araújo et al., 2008; Bolnick et al., 2002). Most of the comparisons of the thermal sensitivity of a given phenotypic trait across individuals under different environmental conditions have been conducted to date on reaction norm descriptors (e.g., comparisons of mean phenotypic differences and cardinal temperatures; Gibert et al., 1998) or degree of plasticity (e.g., regression for linear reaction norms or horizontal [warmer–colder], vertical [faster‐slower], and shape [generalist–specialist] shifts of non‐linear reaction norms; Izem & Kingsolver, 2005; Martin et al., 2011; van de Pol, 2012). Such approaches have proved highly valuable, but may not be suitable for decomposing the overall variation or distinguishing differential responses among populations (Bulté & Blouin‐Demers, 2006) or including intra‐ and interindividual sources of error (Angilletta, 2006) in ANOVA and random regression approaches (Gilchrist, 1995; Lynch & Gabriel, 1987).

One possible complementary approach to the description of variation between reaction norms involves the use of functional ecology to describe significant variations in the degree of individual specialization within populations and species (Garnier & Navas, 2012). The idea is to translate reaction norms by grouping individual reaction norms into “functional groups” (Gitay & Noble, 1997; Violle et al., 2007). Each of these functional groups responds to the environment in its own way (e.g., low‐ or high‐performance specialists), according to a classification system that is not predetermined (i.e., constrained modes of variation). This approach accounts more effectively for patterns of variation in phenotypic plasticity, through the characterization of three functional components: richness, evenness, and divergence (Mason et al., 2005).

This approach is particularly useful for deciphering variation in continuous reaction norms describing performance as a function of temperature (thermal performance curves or TPC; Huey & Stevenson, 1979), and for documenting patterns of thermal adaptation to prevailing local conditions (Kawecki & Ebert, 2004) across a range of environments (e.g., Mitchell & Lampert, 2000). These patterns play an important role in the case of microorganisms impacting ecosystems, human health, and food security (Fisher et al., 2012) as local adaptation to temperature conditions governs their geographic distribution, phenology, and abundance (Kraemer & Boynton, 2017). This results in impacting the expansion ranges of plant pathogens (e.g., Milus et al., 2009; Robin et al., 2017), as well as the onset and severity of disease epidemics (e.g., Ferrandino, 2012).

In plant pathogens, summarizing the individual variance of aggressiveness traits as population‐scale averages (problematic use of single mean species values; Suffert & Thompson, 2018) or phenotyping individuals under a limited set of temperatures when considering variances (generally about three temperatures in thermal biology studies; Dell et al., 2013; Low‐Décarie et al., 2017) has provided useful information about species distribution. This has made it possible to detect signatures of interindividual variation and adaptation within species and populations (Milus et al., 2006). However, this information cannot be used to infer selection driving population dynamics (Lavergne et al., 2010) or to assess the relevant scales of functional diversity (Martiny et al., 2011; Woodcock et al., 2006). Such analyses go well beyond simple comparisons of mean trait values and would require the characterization of entire TPCs and their variation across different scales.

This study explored the extent of variation in thermal responses of a globally distributed wheat pathogen across space (geographic range) and time (local seasonal dynamics), and uncovered the role that adaptation to local environmental conditions (dynamic evolutionary process) plays in generating this diversity. The analysis of the plasticity and variation of thermal sensitivity across individuals, populations, and scales was conducted in the case of *Zymoseptoria tritici* (formerly *Mycosphaerella graminicola*; Steinberg, 2015), the causal agent of one of the most economically important wheat diseases (Septoria tritici blotch or STB; Dean et al., 2012; Fones & Gurr, 2015). Besides its agronomic relevance, we chose to study this fungal pathogen as its aggressiveness traits are empirically known to be temperature‐sensitive (Lovell et al., 2004; Shaw, 1990) and to display interindividual variation (Bernard et al., 2013; Boixel et al., 2019). The duality of the reproduction modes—asexual and sexual, which both contribute to the local level of genetic structure (Singh et al., 2021; Suffert & Sache, 2011)—makes this epidemiological model particularly interesting: (i) Sexual lineages maintain and increase genetic diversity in pathogen populations, through sexual spores that are wind‐dispersed over long distances from wheat residues at the end of each growing season; and (ii) clonal lineages (asexual reproduction) occur within a single field during the course of an epidemic, through asexual spores rain‐dispersed over short distances. Furthermore, *Z*. *tritici* populations present signatures of adaptation to a wide range of contrasted environments over space (globally distributed pathogen across wheat‐growing areas worldwide; Zhan & McDonald, 2011) and time (covering seasonal changes, e.g., from late autumn to early summer in Europe; Suffert et al., 2015). Drawing on previous local adaptation studies conducted by Zhan and McDonald (2011) and Suffert et al. (2015), we designed a sampling scheme to grasp the levels of functional diversity shaping responses of *Z*. *tritici* populations to contrasted environments.

## 2 MATERIALS AND METHODS

### 2.1 Sampling survey design

**FIGURE 1:**
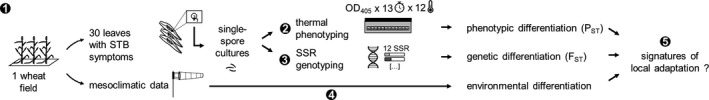
Overview of the methodology for characterizing diversity and adaptive patterns of thermal responses in the sampled *Zymoseptoria tritici* populations. (Step 1) Twelve populations, each composed of either 25 or 30 strains, were collected from diseased leaves in different spatiotemporal locations (8 Euro‐Mediterranean populations collected along geographic thermal gradients and 4 French seasonal subpopulations) with the corresponding mesoclimatic conditions (temperature data). All strains were (Step 2) phenotyped in an *in vitro* growth experiment conducted over a range of 12 temperatures to capture thermal performance curves from growth kinetics involving 13 measurement time points (experimental framework detailed in Boixel et al., 2019); and (Step 3) genotyped for 12 neutral microsatellite (SSR) markers to quantify phenotypic (*P*_ST_) and genetic (*F*_ST_) differentiation. (Step 4) The thermal conditions experienced by individuals over the wheat‐growing season were characterized for each spatiotemporal site. (Step 5) The local adaptation of individuals and populations to temperature was assessed by cross‐comparisons of the spatiotemporal patterns of thermal responses, allele frequency, and thermal conditions

Samples were collected from 12 *Z*. *tritici* populations for the exploration of spatial and temporal components of thermal adaptation (one population being a sample of the complete group of individuals occupying a given wheat plot at a spatiotemporal location) (Figure 1—Step 1). Spatial variation was investigated for 8 populations sampled within the Euro‐Mediterranean region (see detailed sampling information of the geographic scale in Table 1) representative of the contrasting climatic conditions over this large geographic area (covering three—Cfb, Csa, and Dfb—out of seven Köppen–Geiger climate zones in which *Z*. *tritici* is reported as a notable pathogen; Figure S1). One of these sites (Grignon, France) was selected for a comparison of the thermal responses of two pairs of winter and spring subpopulations sampled from neighboring fields, to capture seasonal dynamics over a wheat‐growing season (i.e., over the course of an annual epidemic; see the 4 populations of the seasonal scale in Table 1 and Figure S2). These pairs of subpopulations were subject to seasonal variation from November to February and from March to June, respectively. For each of the 12 populations, we collected 25–30 isolates at random from wheat leaves with STB symptoms, which were placed on a wet filter paper in moist chambers to promote the extrusion of *Z*. *tritici* cirrhi. On each leaf, one cirrhus from a single pycnidium was retrieved for isolation in pure culture (see Methods S1 for more details). After two subculturing for obtaining pure single‐spore strains, *Z*. *tritici* spore suspensions were stored at −80°C in cryotubes, in a 1:1 glycerol–water mixture. Prior to thermal phenotyping, strains were subcultured once just after their thawing. The time elapsed between strain isolation and phenotyping experiments ranged from 1 (Euro‐Mediterranean geographic populations) to 5 (French seasonal subpopulations) years. These conditions reduced the potential effects of previous environmental acclimation (for instance, via transgenerational plasticity or epigenetics), although such effects cannot be completely excluded. The strains were later confirmed to be genetically unique strains based on neutral genetic markers. We chose to consider 25 or 30 strains (i.e., individuals) per population instead of the minimum level of 15 identified on the basis of a rarefaction analysis (Figure S3) for estimating the diversity of thermal responses in *Z*. *tritici* with sufficient power, accuracy, and precision (Dale & Fortin, 2014).

**TABLE 1:** Summary information for the 12 *Zymoseptoria tritici* populations

Scale	ID	Sampling site	Coordinates^a^	Climate^b^	Cultivar	Time window^c^			Sample^d^
Geographic	RU	Russia, Moscow	55.649, 36.958	Dfb	Moskovskaya 56	Spring	June 8, 2016	39–69 (booting–flowering)	30
	KZ	Kazakhstan, Penkovo	54.975, 69.226	Dfb	Lutescens		August 1, 2016		30
	LV	Latvia, Jelgava	56.542, 23.726	Dfb	Zentos		April 28, 2016		30
	DK	Denmark, Flakkebjerg	55.308, 11.388	Cfb	Hereford		May 10, 2016		30
	FR	France, Grignon	48.843, 1.946	Cfb	Soissons		June 14, 2016		30
	IR	Ireland, Carlow	52.860, −6.909	Cfb	JB Diego		July 6, 2016		30
	TN^e^	Tunisia, Manouba	36.923, 9.839	Csa	Karim		April 1, 2016		30
	IS^f^	Israel, Kiryat Tiv'on	32.696, 35.125	Csa	Galil		March 12, 2017		30
Seasonal	WIN1	France, Grignon (field 1)	48.840, 1.945	Cfb	Soissons	Post‐winter	March 1, 2010	24–25 (tillering)	25
	WIN2	France, Grignon (field 2)	48.843, 1.946						30
	SPR1	France, Grignon (field 1)	48.840, 1.945			Post‐spring	June 24, 2010	83–85 (ripening)	25
	SPR2	France, Grignon (field 2)	48.843, 1.946						30

Note

These populations were specifically sampled along geographic (8 Euro‐Mediterranean populations) and seasonal (4 local French winter (WIN) and spring (SPR) subpopulations) scales with contrasting temperature conditions. Strains were collected from naturally infected wheat fields characterized by a spatial location, a climate zone, a wheat cultivar, and sampling conditions (time window and sample size).

^a^ Latitude and longitude in decimal degree format.

^b^ Köppen–Geiger classification: Cfb, temperate oceanic climate; Csa, hot‐summer Mediterranean climate; Dfb, warm‐summer humid continental climate.

^c^ Season, sampling date, and BBCH‐scale coding system for cereal phenological growth stages (Zadoks et al., 1974).

^d^ Number of strains.

^e^ All individuals from a given location were collected from a single pure stand field of a bread wheat cultivar susceptible to STB, except for the TN population, which was sampled from a durum wheat cultivar.

^f^ *Z. tritici* populations were sampled during the 2015–2016 wheat‐growing season, except for the IS population, which was sampled during the 2016–2017 growing season.

### 2.2 Phenotypic variations in thermal responses

Thermal responses were phenotyped by determining the *in vitro* growth rates of the strains in liquid glucose peptone medium (14.3 g L^−1^ dextrose, 7.1 g L^−1^ bactopeptone, and 1.4 g L^−1^ yeast extract) over a 4‐day period at 12 constant temperatures ranging from 6.5 to 33.5°C (6.5, 9.5, 11.5, 14.5, 17.5, 20.0, 22.5, 24.5, 26.5, 28.5, 30.5, and 33.5°C; Boixel et al., 2019) (Figure 1—Step 2). The growth rate *μ* of each strain at each temperature (*n* = 8; independent replicates) was calculated according to the standardized specific experimental framework developed by Boixel et al. (2019), which has been validated to be representative of *in planta* responses with respect to discrimination between “cold‐ and warm‐adapted” individuals. Thermal performance curves (TPCs) describing *in vitro* growth rate as a function of temperature were established by fitting a quadratic function to the temperature–growth rate (or performance *P*) estimates for each strain: *P*(*T*) = *P*_max_ + *Curv*(*T* − *T*_opt_)^2^ where *Curv* is a shape parameter (see Table S1 for more information on the selection process of the model leading to the highest accuracy of performance estimates over the mid‐temperature range). The key properties of TPCs were estimated through thermal traits commonly used to compare thermal sensitivities (Angilletta, 2006; Kingsolver, 2004). We have retained three parameters to describe the shape of these TPCs and quantify their characteristics: first, maximum performance (*P*_max_), which informs on TPC height (“vertical shift” modes of variation); second, thermal optimum (*T*_opt_), which informs on TPC position at the peak performance (“horizontal shift” modes of variation); and third, thermal performance breadth (temperature range over which performance exceeds 80% of *P*_max_; TPB_80_), which informs on the sensitivity of the response to temperature change around *T*_opt_ (“width shift” modes of variation). The estimates of the minimum and maximum temperatures (*T*_min_ and *T*_max_), which define limits of growth, were not retained for further analysis as they fell outside the range of temperatures tested. Differences in thermal responses were assessed in two successive ways: (i) differences in the range and mean values of *P*_max_, *T*_opt_, and TPB_80_, assessed with parametric or nonparametric (depending on whether the assumptions of normality and homoskedasticity were verified) statistical tests for comparing variances and means; and (ii) typological comparisons grouping together TPCs with similar thermal characteristics (functional thermal groups, referred to hereafter as “thermotypes”) based on a K‐means clustering procedure applied to the covariation of *P*_max_, *T*_opt_, and TPB_80_ for all TPCs (Methods S2). This further analysis of TPCs in thermotypes allowed to categorize individuals into five classes of horizontal position of the curve: “highly cold‐adapted” (CA^+^), “cold‐adapted” (CA), “intermediate” (‐), “warm‐adapted” (WA), and “highly warm‐adapted” (WA^+^) that perform better at lower, low, median, high, and higher temperatures, respectively. When in quotation marks here and hereafter, the term “adapted” refers to this higher performance at specific temperature ranges (e.g., cold or warm environment performers) and, at this point, not directly to an assumption about the adaptation to the local environment in which they have been collected. A comparison of the distribution patterns in thermotypes across populations and scales was conducted to detect phenotypic differentiation based on chi‐squared tests on the observed frequency distribution of thermotypes.

### 2.3 Neutral genetic variation and population differentiation

To assess population genetic differentiation, the 350 individuals composing the 12 *Z*. *tritici* populations were genotyped for 12 neutral genetic markers on DNA extracted from 50 mg of fresh fungal material from 5‐day cultures, following SSR amplification and sequencing in one multiplex PCR sample, and allele size annotation (Gautier et al., 2014; Methods S3a) (Figure 1—Step 3). Population structure was inferred with a Bayesian clustering approach under an admixture and correlated allele frequency model implemented in STRUCTURE (Pritchard et al., 2000). The degree and significance of genetic variability within a population (genetic diversity and allele richness) and differentiation between populations (pairwise estimates of Weir and Cockerham's *F*‐statistic—*F*_ST_—and hierarchical analyses of molecular variance—AMOVA) were evaluated with random allelic permutation procedures in GENETIX (Belkhir, 2004) and Arlequin (Excoffier & Lischer, 2010) software (Methods S3b–d).

### 2.4 Characterization of local climates

Air temperature data for the closest weather stations within a mean 30‐km radius of the eight sampling sites were retrieved from archives of global historical weather and climate data, to obtain: (i) monthly averaged values of 1961–1990 climate normals (Norwegian Meteorological Institute, 2019); and (ii) daily data over the sampling year (US National Climatic Data Center NCDC‐CDO, 2019) (Figure 1—Step 4). Temperature conditions of the sampling sites (annual mean temperature and temperature range) and their representativity of climatic conditions encountered at the Euro‐Mediterranean scale are summarized in Figure S1. These variations in climates have been used to detect signatures of *Z*. *tritici* adaptation to its local environment by conducting an analysis of possible correlations between the key thermal traits *P*_max_, *T*_opt_, and TPB_80_ and the representative temperature conditions of the eight sampling sites (monthly averaged values of 1961–1990 climate normals; Figure S4).

### 2.5 Testing for signatures of local adaptation

Two steps were taken to detect genetic and phenotypic signatures of local adaptation underlying the observed differentiation between populations (Figure 1—Step 5). First, the degree of genetic differentiation for the set of neutral markers (*F*_ST_ index; Weir & Cockerham, 1984) was compared with that for phenotypic traits (*P*_ST_ index; Leinonen et al., 2006). This made it possible to infer departures from neutral expectations (Merilä & Crnokrak, 2001), to determine whether thermal traits were under selection rather than subject to genetic drift (Brommer, 2011). *F*_ST_‐*P*_ST_ comparisons were conducted separately for seasonal (on *T*_opt_) and geographic populations (on *T*_opt_ and TPB_80_), on the basis of sensitivity analyses assessing the robustness of the conclusions to variations in the approximation of *Q*_ST_ by *P*_ST_ (Methods S3e). Second, correlations between local climate conditions and *Z*. *tritici* thermal sensitivity were evaluated, to detect signatures of adaptation. Pearson's correlation coefficients and their statistical significance were established for all possible combinations of thermal traits or thermotypic compositions and for the 20 spatiotemporal thermal variables defining the thermal niche of a climatic site.

## 3 RESULTS

### 3.1 Marked interindividual variation in thermal traits at all scales

**FIGURE 2:**
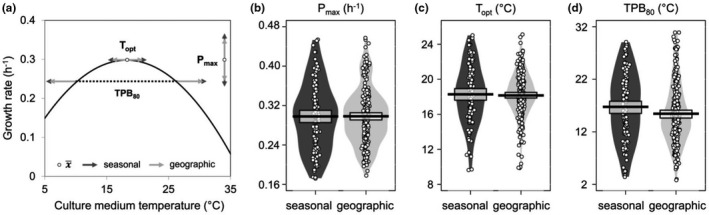
Comparisons of individual variation in *Zymoseptoria tritici* thermal performance curves (TPCs) established for *in vitro* growth rate for the seasonal and geographic scales. (a) The proportion of individual variation around the average TPC for all strains (*n* = 350) is displayed for three key thermal parameters: maximum performance (*P*_max_), thermal optimum (*T*_opt_), and thermal performance breadth (TPB_80_: temperature range over which performance exceeds 80% of *P*_max_). The plot displays the population‐level response (black solid line), the mean value over the 350 individuals for each parameter (open circles and dashed horizontal line), and the spread of the parameter (movement and shift in TPC position along the *x*‐ and *y*‐axes) within the seasonal (*n* = 110) and geographic (*n* = 240) data sets (color‐coded arrows indicating the standard deviation around the mean). The individual variation in TPCs across strains is further broken down into the distribution of (b) *P*_max_, (c) *T*_opt_ and (d) TPB_80_, visualized as their raw individual values (open circles), means (black thick lines), distributions (smoothed density curves), and 95% Bayesian highest density intervals (central rectangular boxes enclosing the means)

We observed a very high level of interindividual variation for the three thermal traits chosen to describe TPCs, within a range of 0.17–0.46 h^−1^ for *P*_max_ (*in vitro* growth rate), 9.6–25.1°C for *T*_opt_, and 2.8–30.9°C for TPB_80_, across all 350 strains. Individual thermal phenotypes are summarized in Figure 2 and available in Dataset S1. The average metapopulation‐level responses in the seasonal and geographic data sets were remarkably similar in terms of their quadratic parameters (Welch's two‐sample *t* test, *p* > .05): *P*(*T*)_seasonal_ = 0.30 − 0.00077 × (*T* − 18.3)^2^ vs. *P*(*T*)_geographic_ = 0.30 − 0.00088 × (*T* − 18.2)^2^. Interindividual variation around this average TPC was greater for the seasonal than for the geographic scale, as demonstrated by the standard shift in TPC position along the *x*‐ and *y*‐axes (Figure 2a) and the distinctly wider density distributions of the three thermal traits at the seasonal scale (Figure 2b–d; Levene's test for homogeneity of variance: *p* = .01 for *P*_max_; *p* < .01 for *T*_opt_; and *p* = .02 for TPB_80_). Interindividual variation within populations was similar at both the geographic and the seasonal scales, with equivalent variances for *P*_max_ ($\bar{x}$ ± 0.06 h^−1^ [SD] on average), *T*_opt_ ($\bar{x}$ ± 2.59°C [SD] on average), and TPB_80_ ($\bar{x}$ ± 5.72°C [SD] on average) within the 12 populations (Levene's test for homogeneity of variance: *p* = .07; 0.51; and 0.13, respectively). The populations may therefore be considered similar in terms of their individual variances for thermal traits. By contrast, they were not similar in terms of the corresponding population means, as significant differences were detected for *T*_opt_ and TPB_80_ (*p* < .05) but not for *P*_max_ (*P*_geographic_ = 0.09; *P*_seasonal_ = 0.75).

### 3.2 A reading grid for functional diversity in individual thermal responses

**FIGURE 3:**
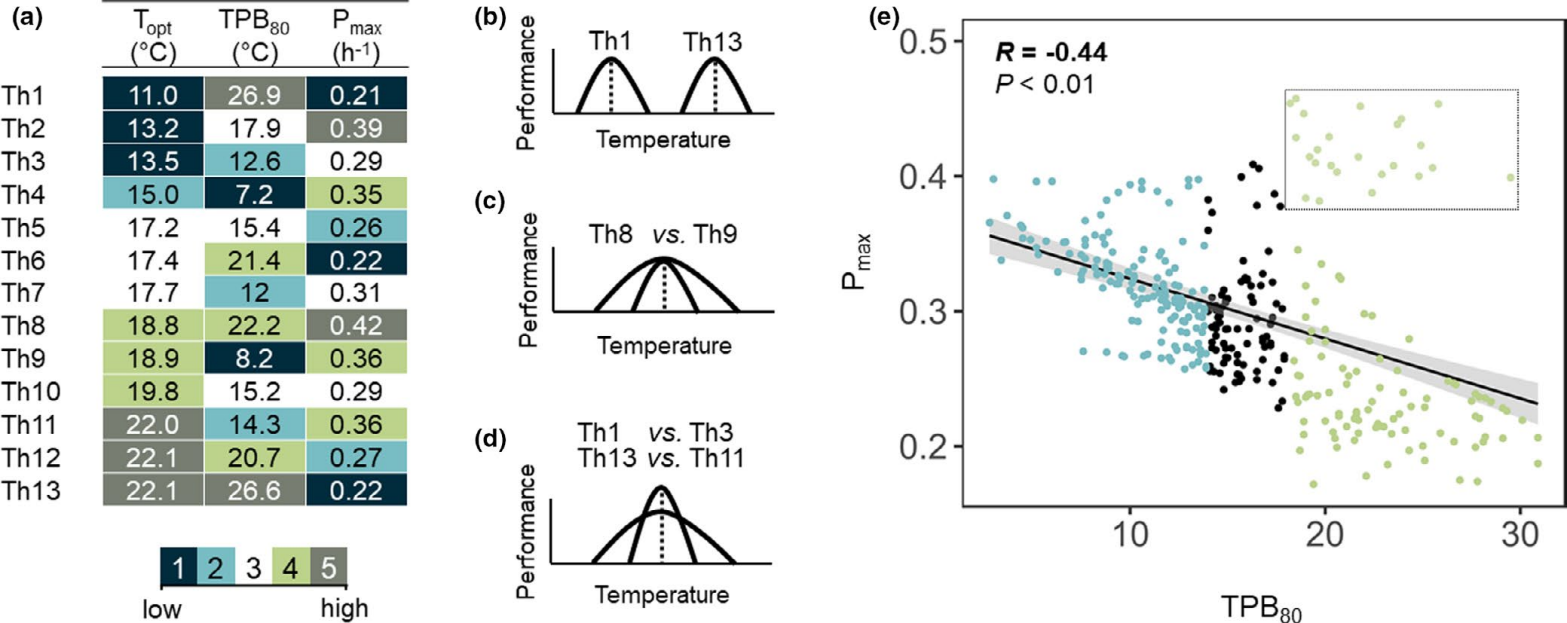
Analysis of the functional differences in thermal performance curves (TPCs) across *Zymoseptoria tritici* strains. (a) Heatmap highlighting the intrinsic features of the 13 *Z*. *tritici* thermotypes (Th) defined on the HCPC clustering of the 350 individual TPCs (see Figure S5). A five‐level scale was defined to summarize the overall difference in low and high values of *P*_max_ (low‐ vs. high‐performance strains); *T*_opt_ (cold‐ vs. warm‐adapted strain); and TPB_80_ (specialist vs. generalist strain): statistically significant (1) much lower, (2) lower, (3) no deviation, (4) higher, and (5) much higher value, relative to the overall mean of each parameter over the whole data set. The indicated *T*_opt_, TPB_80_, and *P*_max_ values correspond to the “barycenter” of each thermotype. (b, c, d) Three common documented shifts in thermal biology studies were identified: (b) a horizontal shift with variations in the position of TPCs along the temperature axis distinguishing “cold‐adapted” vs. “warm‐adapted” thermotypes; a horizontal stretch distinguishing “generalist” vs. “specialist” thermotypes (c) without or (d) with trade‐offs between *P*_max_ and TPB_80_ (TPC axes: *P*: performance; *T*: temperature). (e) Scatter plot highlighting a trade‐off between *P*_max_ and TPB_80_. *P*_max_ is generally negatively related to TPB_80_ except for a group of TPCs with both high TPB_80_ and *P*_max_ (green points surrounded by a rectangle). The regression is displayed as a solid line, with its 95% confidence interval as a shaded area, together with Pearson's correlation coefficient *R* and its *p*‐value *p*

TPCs were classified into thermotypes with similar thermal responses (Hopkins' statistic of 0.71, indicating clustered data and justifying the establishment of such a typology; Methods S2a). The diversity of TPCs encountered in the data set was optimally partitioned into 13 thermotypes (Th1 to Th13; Figure S5), for which relative degrees of temperature specialization were described in terms of the *T*_opt_ (“cold‐ vs. warm‐adapted”), TPB_80_ (“specialist vs. generalist”), and *P*_max_ (“low‐ vs. high‐performer”) dimensions (Figure 3a). These thermotypes illustrated two commonly documented non‐exclusive shifts in TPC along thermal gradients: a horizontal shift (low‐temperature vs. high‐temperature generalists or low‐temperature vs. high‐temperature specialists; e.g., Th1 vs. Th13 in Figure 3b) and a generalist–specialist shift without (Th8 vs. Th9 in Figure 3c) or with (Th1 vs. Th3 or Th11 vs. Th13 in Figure 3d) trade‐offs between *P*_max_ and TPB_80_ (i.e., when one cannot increase without a decrease in the other). Indeed, regression analysis revealed a significant negative correlation between *P*_max_ and TPB_80_ across all individuals (Pearson's correlation coefficient: *R* = −.44; *p* < .01). About 10% of individuals did not follow this pattern, with high values of both *P*_max_ and TPB_80_. These high‐performer generalists (i.e., the strains of Th8) may be considered as “jack‐of‐all‐temperatures” as they perform well over the whole range of temperature covered in the experiment (Huey & Hertz, 1984; Figure 3e). Each cluster included strains from both geographic and seasonal populations (Figure S5), but with an uneven distribution (difference in the Jaccard distance, with a highest pairwise difference of 0.62 between WIN1 and SPR1) and an uneven relative abundance of the 13 thermotypes over the two scales. This relative abundance varied by a factor of up to two for the thermotypes Th5 and Th7. The various thermotypes were not equally distributed across the 12 populations either (chi‐squared test for given probabilities, *p* < .01). This heterogeneous distribution was particularly pronounced for high‐temperature generalists (see the contributions of Th12 and Th13 to the total chi‐squared score for the comparison of distributions across seasonal and geographic populations in Figures S6d and S7c). Four thermotypes together accounted for almost half the entire data set (Th5, Th6, Th7, and Th10). The distinguishing features of these four thermotypes were their average behavior with respect to *T*_opt_ (Th5, Th6, Th7), TPB_80_ (Th5, Th10), and *P*_max_ (Th7, Th10).

### 3.3 Thermal phenotypic differentiation of Euro‐Mediterranean populations

**FIGURE 4:**
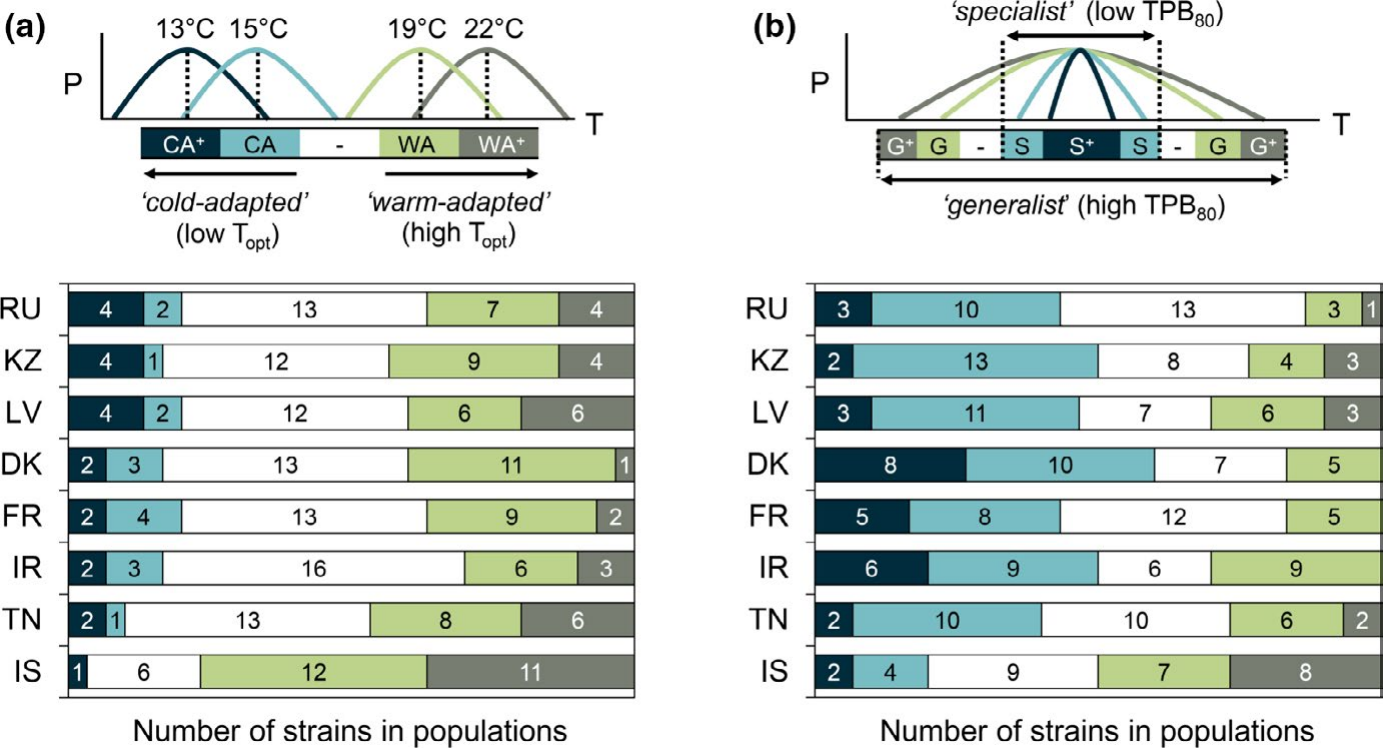
Thermal differentiation in the functional composition of the 8 geographic *Zymoseptoria tritici* populations. The functional composition of these populations is displayed according to two complementary reading grids relating to: (a) optimal temperature, with the relative proportions (*x*‐axis) and corresponding number of individuals (bar values) of “highly cold‐adapted” (CA^+^), “cold‐adapted” (CA), “intermediate” (‐ in white), “warm‐adapted” (WA), and “highly warm‐adapted” (WA^+^) thermotypes within each population; (b) thermal breadth with the relative proportions (*x*‐axis) and corresponding number of individuals (bar values) of high (S) and very high (S^+^) specialist (mean TPB_80_ of 10.8°C) vs. high (G) and very high (G^+^) generalist (mean TPB_80_ of 23.6°C) thermotypes Populations were sampled in RU (Russia), KZ (Kazakhstan), LV (Latvia), DK (Denmark), FR (France), IR (Ireland), TN (Tunisia), and IS (Israel)

For population‐level TPCs, significant variation was observed for thermal trait means for *T*_opt_ (Kruskal–Wallis test, *p* < .01) and TPB_80_ (Kruskal–Wallis test, *p* < .01), but not for *P*_max_ (Kruskal–Wallis test, *p* = .09), for which no population differentiation was detected (Table 2). *P*_max_ values may have been constrained by the upper detection thresholds for optical density (potential saturation of absorbance measurements for individuals with “extreme performance phenotypes”). There was a two‐degree difference in *T*_opt_ between the IS population and the 7 other populations (Table 2). The IS population consisted of individuals performing best at higher temperatures (Figure 4a) with a higher proportion of high‐temperature generalists (Th12 and Th13; 1:3 vs. 1:15 on average for the other geographic populations), accounting for 20.7% of the imbalance in the distribution of thermotypes between populations (see contributions to the total chi‐squared score in Figure S6d). The thermotypes performing better at lower temperatures (CA^+^, Th1‐Th2‐Th3) were particularly abundant in the Dfb populations (RU‐KZ‐LV), as shown by their long‐tailed distributions skewed toward lower temperatures (with 6 highlighted individuals in Figure S6a presenting a *T*_opt_ of about 10.4 ± 0.7°C, i.e., about 7°C below the mean value). The IS population was characterized by a higher TPB_80_ for its average population response than the other populations, particularly DK (19.5 vs. 12.7°C; Table 2). These two populations had opposite patterns in terms of their respective proportions of thermal specialists and generalists (Figure 4b and Figure S6b). More broadly, the individuals with the greatest thermal breadth (G^+^, Th1‐Th13) were less abundant in Cfb populations (DK‐FR‐IR), which were characterized by a higher proportion of more highly specialist individuals (S^+^, Th4, and Th9) than the average (accounting for 10% of the total chi‐squared score; Figure S6d).

**TABLE 2:** Differentiation in the averaged thermal performance curves (TPCs) of the 12 *Zymoseptoria tritici* populations

Thermal trait	Geographic scale								Seasonal scale			
	RU	KZ	LV	DK	FR	IR	TN	IS	WIN1	SPR1	WIN2	SPR2
*P*_max_ (h^−1^)	0.31	0.29	0.29	0.32	0.31	0.28	0.29	0.29	0.31	0.3	0.3	0.29
*T*_opt_ (°C)	17.1 (a)	17.8 (a)	17.8 (a)	17.7 (a)	18.1 (a)	18.2 (a)	18.5 (a)	20.0 (b)	15.9 (α)	20.9 (β)	17.0 (α)	19.3 (β)
TPB_80_ (°C)	15.4 (b)	14.9 (bc)	16.4 (b)	12.7 (c)	14.1 (bc)	14.9 (bc)	15.7 (b)	19.5 (a)	14.8	18.9	17	16.4

Note

Each population is characterized by the population mean values for maximum performance (*P*_max_), thermal optimum (*T*_opt_), and thermal performance breadth (TPB_80_) of individual TPCs. Significant differences in the parameters of TPCs between populations were assessed separately for geographic and seasonal populations, through mean comparisons. The Latin (geographic analysis) and Greek (seasonal analysis) letters in brackets indicate significant differences in post hoc tests.

### 3.4 Seasonal phenotypic shifts within local populations

**FIGURE 5:**
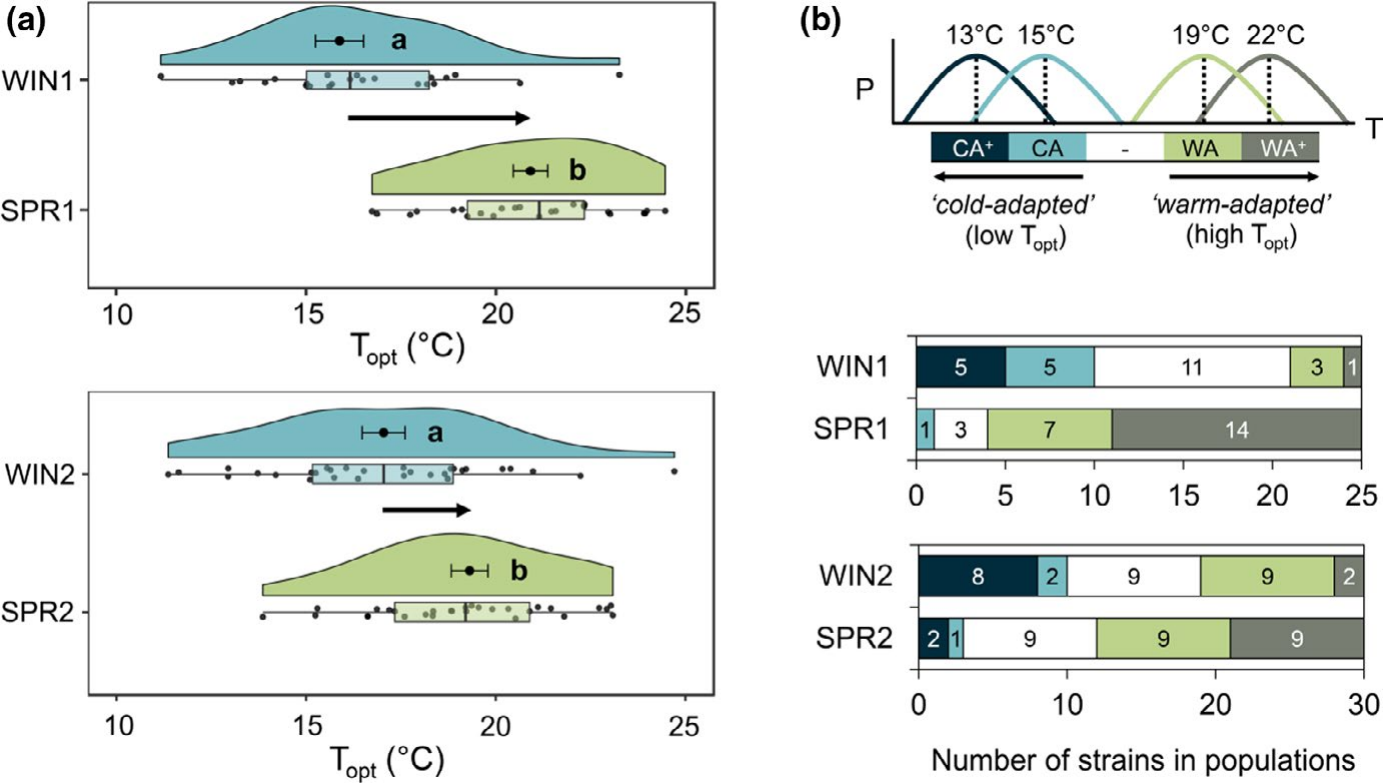
Individual differentiation in the thermal optimum of *Zymoseptoria tritici* strains between French winter and spring subpopulations. (a) The population‐level thermal optima (means ± SEM) are presented together with the distribution of individual *T*_opt_ within populations (associated raw data points, boxplots, and split‐half violins). A significant shift in *T*_opt_ distribution along the temperature axis was detected between winter (WIN1 and WIN2) and spring (SPR1 and SPR2) subpopulations sampled from two local neighboring fields (annotated 1 and 2). The letters indicate the output of paired Student's *t* tests with *p* < .05. (b) Functional thermotype composition within winter and spring subpopulations is displayed as relative proportions (*x*‐axis) and corresponding numbers of individuals (bar values) for “highly cold‐adapted” (CA^+^), “cold‐adapted” (CA), “intermediate” (‐ in white), “warm‐adapted (WA), and “highly warm‐adapted” (WA^+^) thermotypes

Spring subpopulations (SPR1 and SPR2) had a higher thermal optimum than winter subpopulations (ANOVA, *p* < .01), with a horizontal shift of *T*_opt_ toward higher temperature of the order of 5°C (SPR1) and 2.3°C (SPR2) on average (Table 2 and Figure 5a). In terms of thermotype composition, these two pairs of subpopulations differed principally in their relative proportions in strains that perform better at warmer temperatures (WA^+^). WA^+^ strains were significantly more abundant in SPR populations (Figure 5b) than in WIN populations, accounting for 33.4% of the total chi‐squared score for difference in thermotype distributions between WIN and SPR populations. Conversely, WIN populations had a higher proportion of individuals performing better under colder conditions (CA^+^; Figure S7).

### 3.5 Signatures of local adaptation to mean annual temperature conditions

**FIGURE 6:**
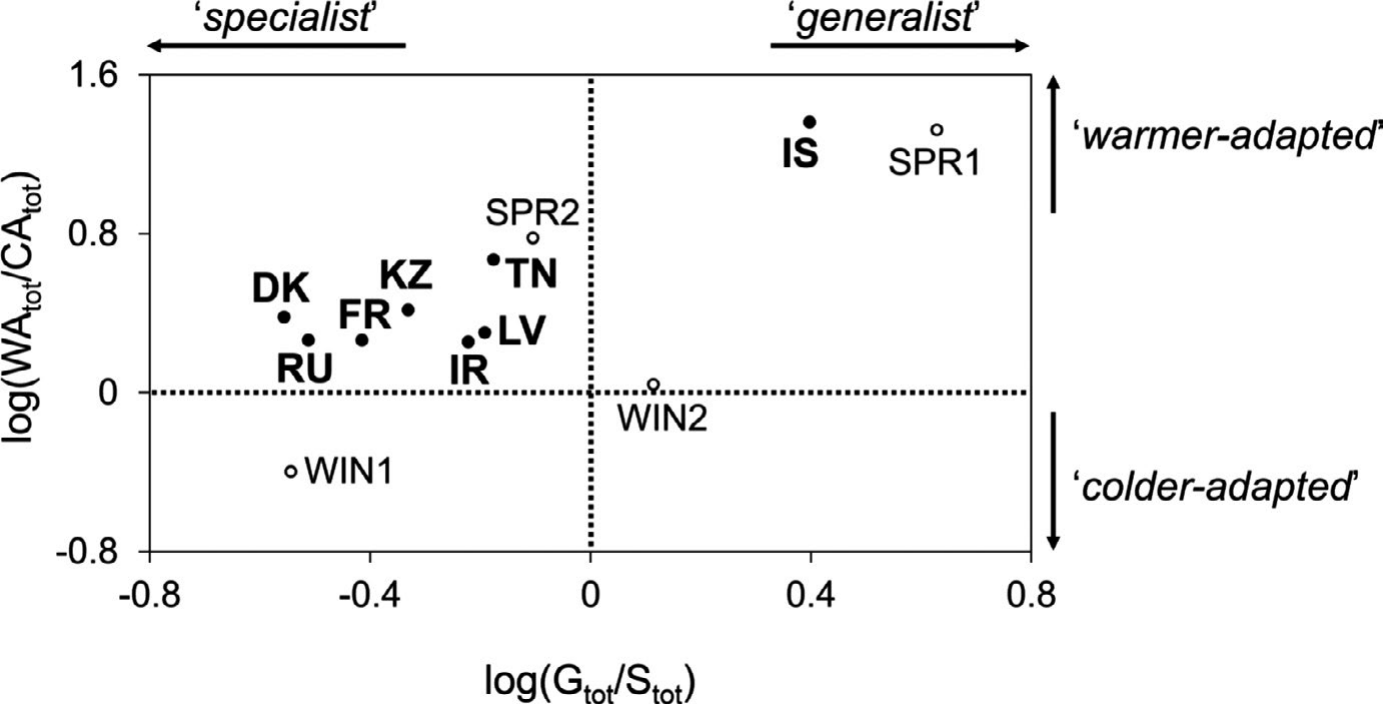
Functional diversity in thermal responses between the 12 *Zymoseptoria tritici* populations. Geographic (in bold) and seasonal (in standard text) populations are situated along: (i) a scale of increasing degree of adaptation to warm conditions (*y*‐axis) discriminating colder‐ and warmer‐adapted populations (logarithm of the ratio of the total of “warm‐adapted” individuals—WA and WA^+^—to the total of “cold‐adapted” individuals—CA and CA^+^); (ii) a scale of thermal breadth continuum (*x*‐axis) discriminating more specialist and more generalist populations (logarithm of the ratio of the total number of generalist individuals—G and G^+^—to the total number of specialist individuals—S and S^+^)

**FIGURE 7:**
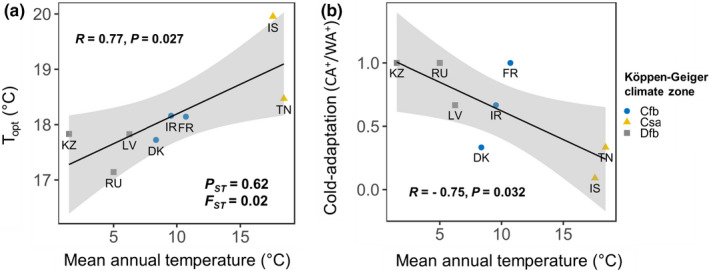
Signatures of *Zymoseptoria tritici* adaptation to the mean annual temperature of the local environment in the 8 geographic populations. (a) Relationship between population thermal optimum (*T*_opt_) and the mean annual temperature of the sampling sites (monthly averaged values of 1961–1990 climate normals, themselves positively correlated with the monthly averaged values over the sampling year; Pearson's correlation coefficient: *R* = .98, *p* < .01). Population differentiation in *T*_opt_ relative to neutral genetic differentiation is indicated by *P*_ST_ and *F*_ST_ values. (b) Relationship between cold adaptation level, defined as CA^+^/WA^+^ (ratio of the number of “highly cold‐adapted” to “highly warm‐adapted” thermotypes), and the mean annual temperature at the sampling site. Linear dependence between these pairs of variables is indicated by the regression line (solid line and its 95% confidence interval, shown as a shaded area), Pearson's correlation coefficient *R*, and its associated *p*‐value *p* (see Figure S1 for a description of the three Köppen–Geiger climate zones, Cfb, Csa, and Dfb)

Neutral molecular markers revealed that all strains were genetically different. We observed no difference in the genetic structure of the 12 populations, with similar allele frequencies at each locus (Figures S8, S9 and Table S2), suggesting a constant mixing of populations through substantial continental gene flow, as underlined in previous studies for *Z*. *tritici* (Boeger et al., 1993). The partitioning of genetic variance assessed by a hierarchical analysis of molecular variance (AMOVA; Table S3) indicated that within‐population variation accounted for most of the molecular variance (99.4%), with much lesser amounts among populations (0.6%). In particular, there was a positive but nonsignificant correlation between genetic and geographic distance among populations (*r* = .22, *p* = .08; Figure S10). Evidence of local adaptation (Figure 6) was detected with the occurrence of a robust *P*_ST_–*F*_ST_ difference for the *T*_opt_ of both geographic and seasonal populations and for the TPB_80_ of geographic populations (Figure 7 and Figure S11). An analysis of possible correlations between these thermal traits and the temperature conditions of the eight sampling sites (monthly averaged values of 1961–1990 climate normals) indicated that the mean thermal optimum of geographic populations increased with mean annual temperature (Figure 7a). The level of cold adaptation of these populations (measured as the ratio of highly “cold‐adapted” to highly “warm‐adapted” strains) was negatively and significantly correlated with the same climatic variable (Figure 7b).

## 4 DISCUSSION

### 4.1 Thermal phenotyping of *Zymoseptoria tritici* strains beyond the usual tests of “thermal sensitivity”

As several other studies on thermal phenotyping (Birgander et al., 2018; Paisley et al., 2005; Robin et al., 2017; Stefansson et al., 2013; Zhan & McDonald, 2011), the high‐throughput method used in this study was a standardized *in vitro* approach. By characterizing the TPCs of *Z*. *tritici* strains collected over different spatiotemporal scales, we were able to develop a fine description of the extensive interindividual variation in thermal sensitivity: maximum performance (*P*_max_), thermal optimum (*T*_opt_), and thermal performance breadth (TPB_80_). This detailed characterization was made possible by the large range of temperatures and the high resolution of this experimental study (12 temperatures, ranging from 6.5 to 33.5°C), the extensive sampling strategy (350 strains from 12 populations collected within the Euro‐Mediterranean region), and the use of a dedicated and previously validated experimental framework based on turbidity measurements (Boixel et al., 2019). It is important to bear in mind that these turbidity measurements may not reflect the sole growth multiplication rate *via* yeast‐like budding but more precisely quantify the total fungal biomass that could be affected by the pleomorphic nature of some strains of *Z*. *tritici* under some environmental stimuli (e.g., partial transition to pseudohyphae or induction of a few chlamydospores, a very recently highlighted form at high temperatures; Francisco et al., 2019). Precautions were taken to work under culture conditions limiting morphological transitions in the 4‐day time window of the experiments: Very few hyphae were observed at 96 h when validating the method (see ESM1‐3 in Boixel et al., 2019). As such, this framework enables to detect differences in thermal sensitivity between isolates (whatever the physiological bases that underpin these differences) and to go beyond the usual tests of “thermal sensitivity” based on two temperatures, which can be misleading due to the non‐linearity of reaction norms (Angilletta, 2009). An advantage of the *in vitro* approach is that it enables large‐scale investigations while alleviating major confounding factors (e.g., cross‐effect between host resistance and temperature adaptation; Pariaud et al., 2009). It should be mentioned, however, that, in contrast to *in vitro* responses, *in planta* processes exhibit narrower temperature responses and shifts to lower thermal optima (Boixel et al., 2019; Chaloner et al., 2020). Despite this *in planta* restriction of temperature niche breadth, a ranking consistency of thermal sensitivity between “cold‐ and warm‐adapted” strains, consistent with the concept of phenotypic integration (Pigliucci, 2003), has been reported in previous studies (e.g., Paisley et al., 2005), notably in the case of *Z*. *tritici* (Boixel et al., 2019; Zhan et al., 2016). This alteration of thermal responses related to disease *in planta* may be due to suboptimal resource conditions (e.g., interaction with the host plant, stress responses, and nutrient restriction) compared with growth in axenic culture (Chaloner et al., 2020).

### 4.2 Geographic variation in thermal response among *Zymoseptoria tritici* populations

The geographic variation of TPCs provides evidence of thermal adaptation to local conditions in *Z*. *tritici*, with: (i) an increase in the mean thermal optimum of a given population with the annual mean temperature of its location of origin; (ii) a particularly marked adaptation to high temperatures of the population sampled in Israel, consistent with the results obtained for another Israeli population investigated by Zhan and McDonald (2011); and (iii) differences in the level of specialization of individuals between populations with higher proportions of specialist individuals in the Cfb (climatic zone with lower annual temperature range) than in the Dfb (climatic zone with higher annual temperature range) populations, consistent with the assumption that thermal generalists are favored in more variable environments. By contrast, over a smaller geographic scale (France), using the same experimental method, we detected (i) high levels of local diversity but no structure of thermal responses between spring populations sampled along a gradient of increasing mean annual temperature; and (ii) a marked difference between post‐winter populations sampled along a gradient of increasing annual temperature range: the presence of thermal generalists in the population exposed to the largest annual temperature range (19.9°C) vs. the complete absence of such generalists in the population exposed to the smallest annual temperature range (11.9°C; Boixel et al., 2019). The phenotypic differentiation of thermal responses at the population level probably results from local short‐term selection of the fittest strains over the course of an annual epidemic. We investigated the adaptation to the location of origin of populations with respect to mesoclimatic temperature conditions. The patterns of adaptation detected may have been blurred by a non‐optimal descriptive resolution of the thermal niche. Indeed, the microenvironment actually perceived by organisms can diverge from the surrounding macroenvironment due to complex biophysical filters across scales (here phylloclimate vs. mesoclimate; Chelle, 2005). Scaling the actual climate perceived by *Z*. *tritici* populations down to the phylloclimate would help refining the definition of a thermal niche for each population (Pincebourde & Casas, 2019; Pincebourde & Woods, 2012). Such an approach might provide deeper insight into the maintenance of high levels of diversity and some degree of maladaptation in individual thermal responses within each population.

### 4.3 Seasonal dynamics of thermal responses in two local *Zymoseptoria tritici* populations

The interindividual variation of thermal traits was conserved across populations (similar variance within populations) but was generally more marked over the seasonal scale (for a similar average metapopulation‐level response between seasonal and geographic scales). These findings are particularly striking because the choice of geographic populations made it possible to cover three contrasting Köppen–Geiger climatic zones (Figure S1). Sampling over the geographic scale occurred during spring, between the two time points investigated at the seasonal scale (i.e., post‐winter and post‐spring conditions). These seasonal samplings highlighted a marked seasonal shift of TPCs toward higher temperatures and changes in the thermotype composition of two local *Z*. *tritici* populations. This result is consistent with previous observations of seasonal short‐term selection on aggressiveness traits (Suffert et al., 2015). This study thus reveals a two‐tier thermal adaptation, with seasonal dynamics nested within and potentially occurring in each geographic local adaptation over annual epidemics. This key finding shows that adaptive patterns are “eco‐evolutionary snapshots” that should be interpreted with caution, to such an extent that certain evolutionary dynamics of microbial populations can be of one type over a very short timescale and another type over longer timescales. Indeed, adaptive dynamics may differ with the timescale investigated (annual or pluriannual), particularly for annual crop pathogens with both sexual and asexual reproduction cycles, such as *Z*. *tritici* (Suffert et al., 2018). Our findings could be summarized by the counterintuitive statement “local seasonal adaptation is stronger but more fleeting than geographic adaptation” although we would expect that regions with lower seasonal contrasts in temperature (e.g., with mild winters) will exert weaker selective pressure. The use of sequential temporal sampling would make it possible to capture shifts in thermal adaptation over and between wheat‐growing seasons and to detect potential trade‐offs between aggressiveness and survival over winter (e.g., Montarry et al., 2007).

### 4.4 From adaptation patterns to eco‐evolutionary processes

Consistent with previous studies, our findings highlight the existence of high levels of genetic diversity and an absence of its structuring across *Z*. *tritici* populations collected from local wheat fields (Zhan et al., 2001) up to the regional and continental scales (Linde et al., 2002; Schnieder et al., 2001) or over the course of an epidemic cycle (Chen et al., 1994; Morais et al., 2019). The high level of gene flow suggested by this low level of genetic differentiation between populations may partly explain the maintenance of some degree of maladaptation to local conditions (e.g., the detection of three CA+individuals in the IS population). More generally, we observed almost all the “*T*_opt_‐adapted” thermotypes (CA+, CA, WA, WA+) in each phenotyped population (except that CA individuals were absent from the IS population and CA+ individuals were absent from the SPR1 population), despite the clear patterns of adaptation observed for *T*_opt_ and the large differences in environmental temperatures. This maintenance of diversity suggests that *Z*. *tritici* is highly tolerant to thermal variations (high probability that environmental conditions are favorable to the development of at least some individuals in a given local population). One possible explanation for this finding is that the substantial adaptation of populations to their environments (e.g., only “warm‐adapted” individuals under a warm environment) is hindered by a balance between gene flow and local selection (Ronce & Kirkpatrick, 2001). It also raises the issues of the occurrence of counter‐selection during the interepidemic period that might explain how local populations shift in thermotype structure to reestablish similar structures between years through heritability and genetic reassortment during sexual reproduction, which is driven by antagonistic density‐dependent mechanisms (Lendenmann et al., 2016; Suffert et al., 2019). Further studies are required to determine the extent to which the detected pattern of geographic adaptation is driven by the thermal conditions of the environment. For this, the potential counteracting effects of selection, gene flow, random genetic drift, mutation, and recombination on the increase or decrease in genetic variation would need to be assessed (Hanson et al., 2012). In particular, the combination of the high diversity of thermal responses in *Z*. *tritici* highlighted here, their heritability (Lendenmann et al., 2016), and the high level of local heterogeneity within wheat canopies (Chelle, 2005) suggests that local thermal conditions probably exert strong selection pressure on thermal sensitivity (for which TPCs are probably the best proxy as they may themselves be direct targets of selection; Scheiner, 1993; Via, 1993), even in the presence of high gene flow due to long‐distance ascospore migration (hundreds to thousands of km). The comparison of population genetic divergence for neutral marker loci (*F*_ST_) exemplified the extremely high diversity of *Z*. *tritici* populations, even at a very local scale and whatever their putative admixture and/or local adaptation characteristics, in line with previous population genetic studies (e.g., Linde et al., 2002; Morais et al., 2019; Singh et al., 2021), including one on thermal adaptation (Zhan & McDonald, 2011). The *F*_ST_ values observed here and in other studies of *Z*. *tritici* are low compared with the reported mean *F*_ST_ in published studies of genetic diversity in fungi (*F*_ST_ = 0.2); a mean value that hides substantial differences in dispersal abilities and/or effective population sizes between fungal pathogens with reported data from low (*F*_ST_ = 0.02) to very high (*F*_ST_ = 0.9) genetic differentiation (Giraud et al., 2008; Morjan & Rieseberg, 2004).

### 4.5 Functional group composition: an operational approach for investigating population dynamics

**FIGURE 8:**
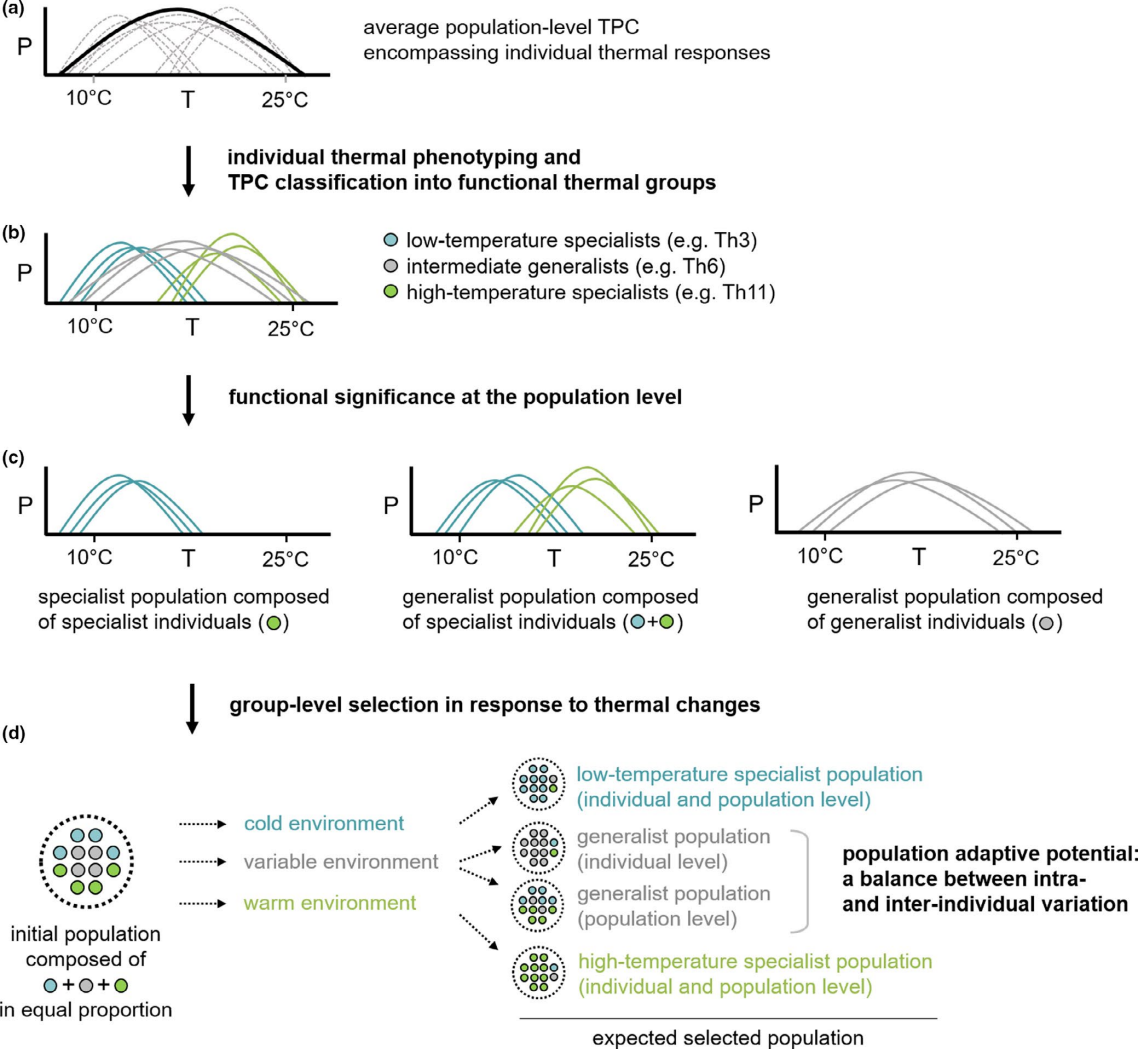
Summary of the way categorization into thermotypes (functional thermal groups) sheds light on the translation of population diversity patterns into selection dynamics in response to climate conditions. (a) Average population‐level TPC (solid line) concealing a set of varied individual TPCs (dashed lines); (b) breakdown of the variation in individual TPCs based on their classification into thermotypes and screening for a functional significance of variation at the individual level (given example of three thermotypes within which individuals are considered functionally redundant: low‐temperature specialists, intermediate generalists, and high‐temperature specialists). (c) Categorization tackling functional redundancy at the population level (i.e., whether the thermotypes composing a population are more or less well differentiated within the whole functional space). The three populations presented here demonstrate the relevance of considering functional redundancy vs. vacant functional space when assessing emergent properties of populations such as a generalist nature at population level (e.g., a generalist population can be composed of specialist individuals with narrow individual TPB_80_ distributed over the functional space, resulting in broad TPB_80_ population coverage). (d) The translation of populations into functional groups makes it possible to investigate group‐level selection, testing for general assumptions of adaptation to given environments (e.g., competitive advantage of low‐temperature specialists in cold environments, generalists in variable environments, and high‐temperature specialists in warm environments) providing insight into the potential of populations to adapt to changes in their environment (a subtle balance between diversity levels for intra‐ and interindividual variation in thermal responses)

Our study illustrates how the functional classification of TPCs into thermotypes with multivariate statistical procedures can provide a complementary means of deciphering diversity patterns in the biological responses quantified in reaction norms. In particular, it constitutes an operational tool for assessing functional similarity at the individual level (i.e., whether the apparent variation observed in thermal parameters is functionally significant; Figure 8a,b) and at the population level (i.e., whether the thermotypes constituting a population are more or less well differentiated within the whole functional space; Figure 8c). However, caution will be required in the extension of this approach to comparisons over multiple data sets, through the development of comparable classification systems, taking into account the variation of the classification with the populations sampled by explicitly stating which ranges of trait values are hidden behind a given group description (e.g., affixing levels of “adaptation” in the sense of higher performance at given temperature ranges: very low, low, high, very high). This description of populations in terms of functional groups makes it possible to move from a description of phenotypic patterns and shifts in population composition to an inference process. This process may, for example, be based on comparisons of the competitive advantage of thermotypes under given thermal scenarios: for example, “do more variable environments favor thermal generalists?” or “is there a shift in the optimal range of thermal responses with mean temperature conditions?” (Figure 8d). This classification into thermotypes enabled here to go beyond a purely descriptive framework, and future investigations will need to be undertaken to tackle the physiological bases of these differentiations in thermal responses. The thought‐provoking results of Francisco et al. (2019) could be used to test whether several strains belonging to those thermotypes also correspond to specific or main morphotypes that would increase their tolerance under some environmental conditions (e.g., if “warm‐adapted” individuals exhibit higher proportions of stress‐tolerant growth forms such as chlamydospores under warmer temperatures). All in all, this functional approach lays the foundations for future studies of the potential of pathogen populations to adapt to changes in their environment, from seasonal changes in the short term, to global warming in the long term. In particular, it will prove useful in gaining a fuller understanding of how new aggressive fungal strains may emerge and expand into previously unfavorable environments (Milus et al., 2009; Mboup et al., 2012; Stefansson et al., 2013). This is a crucial area of investigation that is all too often overlooked in models for predicting plant disease epidemics in conditions of climate change (West et al., 2012).

## 5 CONCLUDING REMARKS

The detailed characterization of a microbial phenotype as a profile rather than a mean allowed for analyses that accounted for the range of sensitivities of individual strains rather than solely their mean sensitivity. This gave insight into the high level of functional divergence in the plasticity and variation of individual thermal responses over geographic and seasonal scales, highlighting the occurrence of two‐tier dynamics in thermal adaptation. These findings raise intriguing questions regarding the mode of selection operating on these functional groups of individuals with similar competitive advantages in given thermal conditions. Deciphering the mechanisms underlying this maintenance of diversity in population phenotypic composition will prove useful for expanding our understanding of eco‐evolutionary responses and the potential of populations, species, and communities to adapt to environmental change.

## CONFLICT OF INTEREST

The authors declare no conflict of interest.

## AUTHOR CONTRIBUTIONS

**Anne‐Lise Boixel:** Conceptualization (equal); data curation (lead); formal analysis (lead); methodology (equal); software (lead); visualization (lead); writing – original draft (lead); writing – review and editing (equal). **Michaël Chelle:** Conceptualization (equal); funding acquisition (equal); investigation (equal); methodology (equal); project administration (equal); supervision (equal); writing – original draft (supporting); writing – review and editing (equal). **Frédéric Suffert:** Conceptualization (equal); funding acquisition (equal); investigation (equal); methodology (equal); project administration (equal); supervision (equal); writing – original draft (supporting); writing – review and editing (equal).

## Supporting information

Supplementary Material

## Data Availability

The thermal phenotyping data set associated with this manuscript is available on the INRAE Dataverse repository (https://data.inrae.fr/dataset.xhtml?persistentId=doi:10.15454/FZ9NN6).
