# Supplementary material for: Patterns of thermal adaptation in a globally distributed plant pathogen: Local diversity and plasticity reveal two‐tier dynamics
Source: Ecol Evol. 2022 Jan 26;12(1):e8515. doi: 10.1002/ece3.8515 (PMC8796916; doi:10.1002/ece3.8515)
Supplement: Supplementary file 1 — Supplementary Material [file ECE3-12-e8515-s001.docx]

## Supporting Information

**Patterns of thermal adaptation in a globally-distributed plant pathogen: local diversity and plasticity reveal two-tier dynamics (Boixel, Chelle, and Suffert)**

The following Supporting Information is available for this article:

**FIGURES**

- **Fig. S1** Selection of the sampling sites in the Euro-Mediterranean wheat-growing area
- **Fig. S2** Selection of the seasonal subpopulations
- **Fig. S3** Appropriate sample size for estimating diversity in TPCs within *Z. tritici* populations
- **Fig. S4** Characterisation of the thermal niche at each sampling site
- **Fig. S5** Clustering of *Z. tritici* strains into 13 thermotypes
- **Fig. S6** Distribution of thermotypes across the eight Euro-Mediterranean *Z. tritici* populations
- **Fig. S7** Distribution of thermotypes across the four French seasonal *Z. tritici* subpopulations
- **Fig. S8** Genetic diversity and population structure of the 12 *Z. tritici* populations
- **Fig. S9** Delta K plot representing the most probable number of genetic groups (K = 3)
- **Fig. S10** Correlation between genetic and geographic distance among populations
- **Fig. S11** Sensitivity analyses for the robustness of P_ST_–F_ST_ comparisons

**TABLES**

- **Table S1** Selection of a candidate mathematical model for establishing TPCs
- **Table S2** Population-pairwise genetic distance (matrix of F_ST_-values)
- **Table S3** Hierarchical analysis of molecular variance results (AMOVA)

**METHODS**

- **Methods S1** Procedure for the sampling, collection and recovery of *Z. tritici* strains
- **Methods S2** Definition of *Z. tritici* ‘thermotypes’ (functional thermal groups)
- **Methods S3** Procedure for acquiring and analysing multilocus genotypic data

**ADDITIONAL REFERENCES**

**
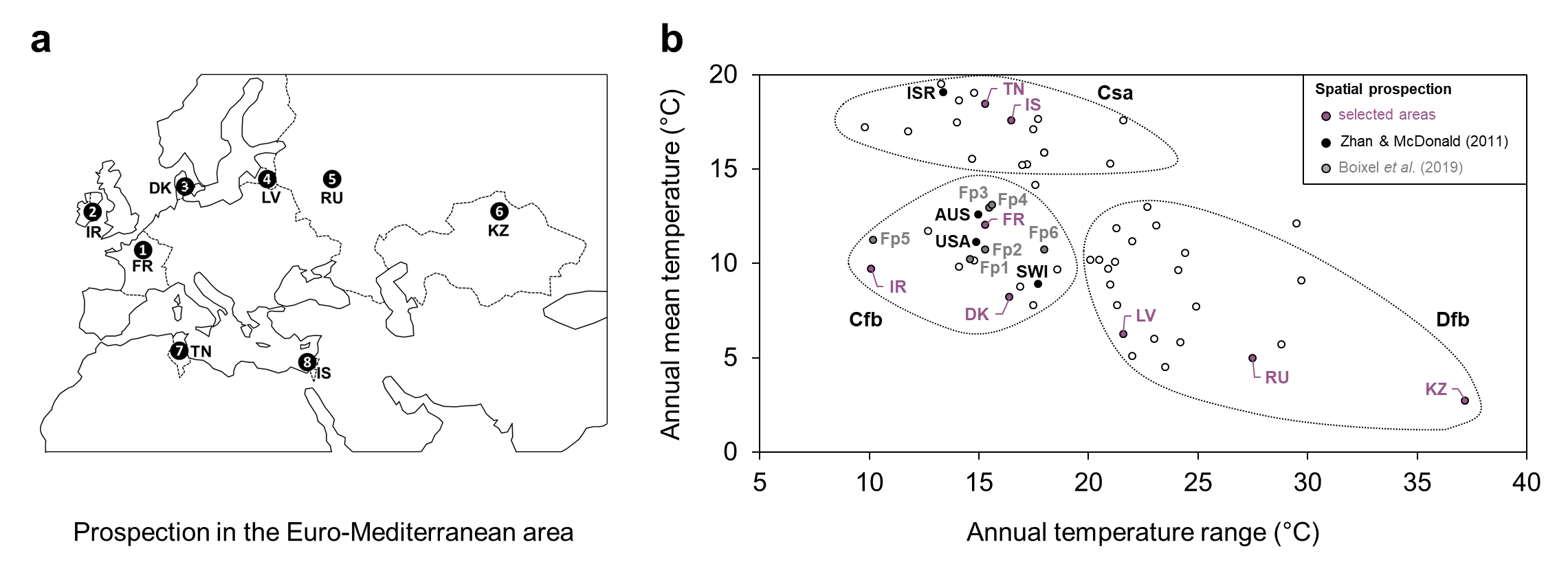
Fig. S1** **Selection of sampling sites in the Euro-Mediterranean wheat-growing area.** (a) Location of the eight fields from which the Euro-Mediterranean populations of *Zymoseptoria tritici* were collected: FR (*Thiverval-Grignon*, France), IR (*Carlow*, Ireland), DK (*Flakkebjerg*, Denmark), LV (*Jelgava*, Latvia), RU (*Moscow*, Russia), KZ (*Penkovo*, Kazakhstan), TN (*Manouba*, Tunisia), IS (*Kiryat-Tivon*, Israel). Dotted lines correspond to the national borders of the countries from which the populations were collected. (b) Representative panel of climatic conditions encountered at the Euro-Mediterranean scale, for classification of the diversity of thermal responses in *Z. tritici* populations. Sampling locations (in purple) were chosen on the basis of 1961-1990 climate normals to maximize environmental heterogeneity at a level much greater than in two previous studies on thermal adaptation in *Z. tritici* (Zhan & McDonald, 2011; Boixel *et al.*, 2019). The scatter plot depicts the annual mean temperature and temperature range (Norwegian Meteorological Institute, 2019) of each country location in the Euro-Mediterranean zone and in the two aforementioned studies (see open, grey and black points, respectively). The various sampling sites were classified according to their predominant Köppen-Geiger climate types (Köppen, 1936; Peel *et al.*, 2007): Cfb (temperate oceanic climate), Csa (hot-summer Mediterranean climate), Dfb (warm-summer humid continental climate). AUS (*Wagga Wagga*, Australia), ISR (*Nahal Oz*, Israel), SWI (*Berga Irchel*, Switzerland) and USA (*Corvallis*, United States) correspond to the sampling locations of the five *Z. tritici* populations investigated in Zhan & McDonald (2011). Fp1 (*Villers-lès-Cagnicourt*), Fp2 (*Versailles*), Fp3 (*Bergerac*), Fp4 (*Lectoure*), Fp5 (*Ploudaniel*) and Fp6 (*Bretenière*) correspond to the sampling locations of the six French *Z. tritici* populations investigated in Boixel *et al.* (2019).


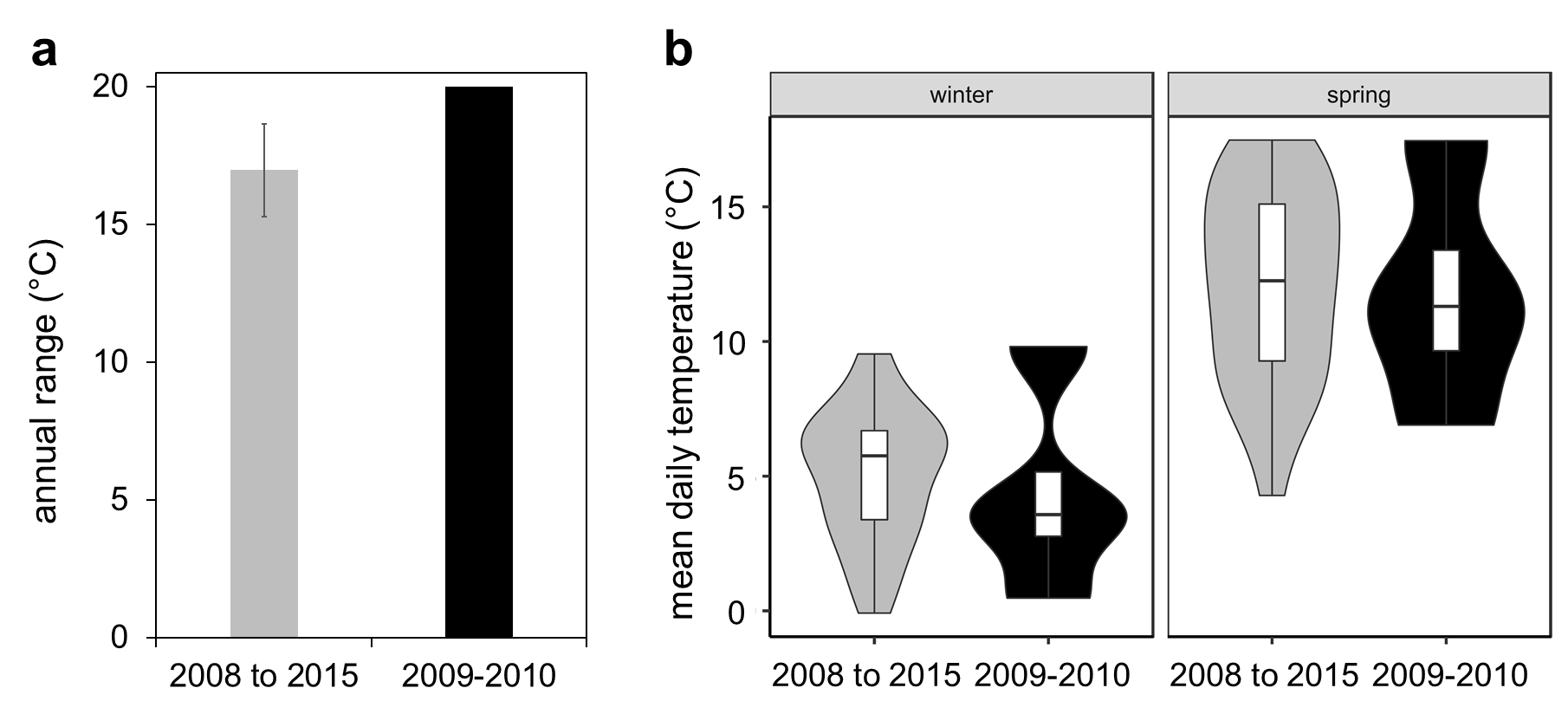


**Fig. S2 Selection of the seasonal subpopulations.** Analysis of the climatic data for *Thiverval-Grignon* from 2008 to 2015 (INRAE AgroClim, 2019) used to select the most contrasting wheat growing season to be investigated from those for which samples were available from the INRAE BIOGER *Z. tritici* collection. Seven pairs of subpopulations were sampled from two neighbouring field plots during a long-term population survey (partially presented in Suffert *et al.*, 2018) over seven wheat growing seasons (from 2008-2009 to 2014-2015): one post-winter subpopulation (sampled at the beginning of March) and one post-spring subpopulation (sampled at the end of June). We investigated seasonal effects in the 2009-2010 subpopulations, which had (a) the highest annual amplitude (20°C, vs. 16.9°C ± 1.7°C in the other seasons) and (b) the greatest contrast between ‘winter’ (November – February: tighter distribution of mean daily temperatures) and ‘spring’ (March – June: shortened low end of the distribution tail of mean daily temperatures) temperature conditions.


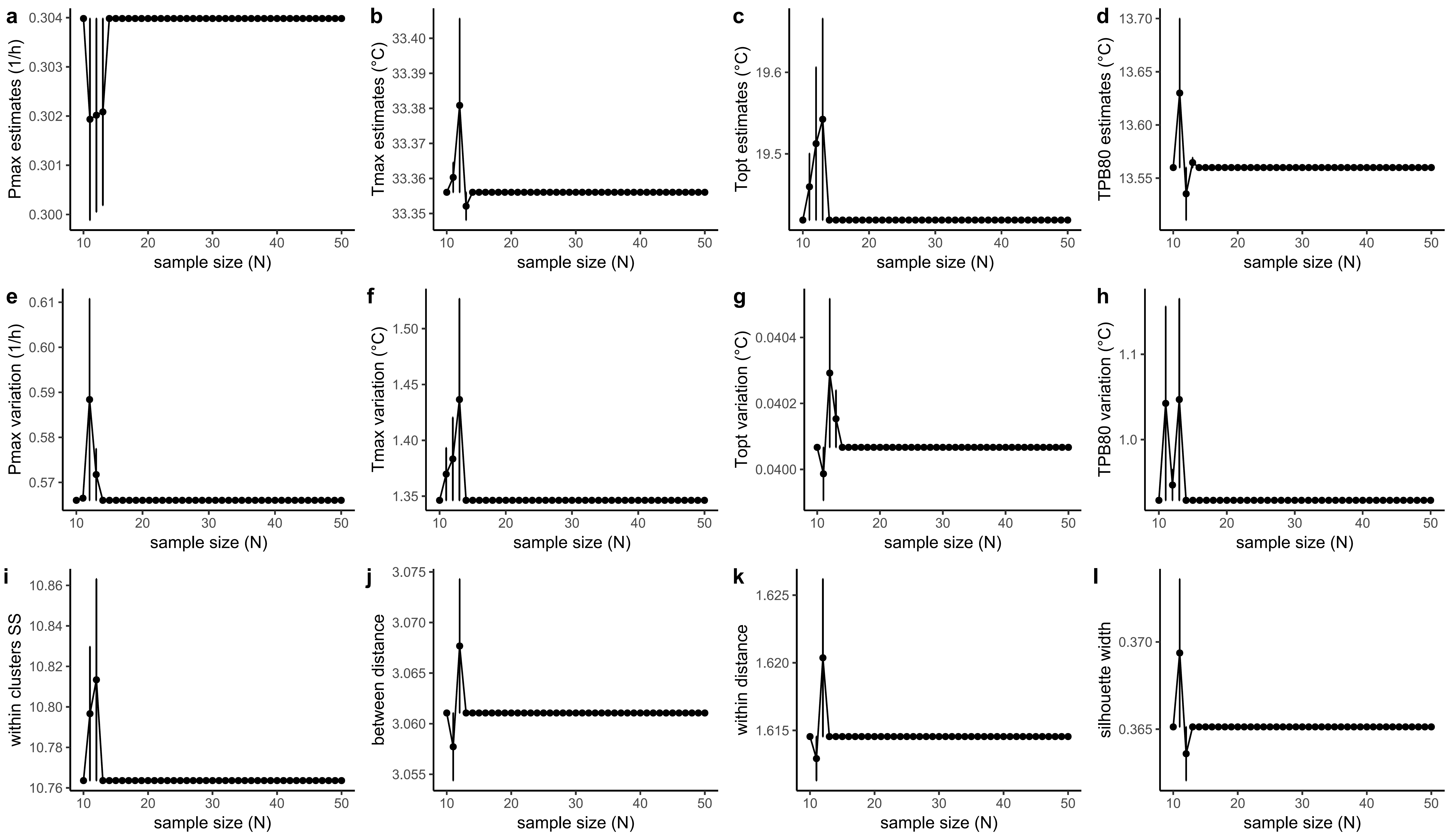


**Fig. S3 Appropriate sample size for estimating diversity in TPCs within *Z. tritici* populations.** For the definition of a target sample size per population, we performed a rarefaction analysis on a set of 66 *Z. tritici* strains that had undergone preliminary phenotyping for their responses to temperature (see Boixel *et al.*, 2019). Rarefaction curves of expected diversity for key thermal parameters (a: mean maximum performance P_max_; b: mean maximum temperature T_max_; c: mean thermal optimum T_opt_; d: mean 80% thermal performance breadth TPB_80_; e: P_max_ standard deviation; f: T_max_ standard deviation; g: T_opt_ standard deviation; h: TPB_80_ standard deviation) and in the typology of thermal responses based on HCPC clusters (i: within-thermotype sum of squares; j: mean distance between clusters; k: mean distance within clusters; l: mean silhouette width providing information about the compactness, separation, and connectivity of the cluster partitions) were obtained for 41 levels of sampling depth (mean ± resampling standard error; *n* = 15 subsampling repetitions per sampling depth) ranging from 10 to 50 individuals per subsample (sample size *N; x*-axis). Similar results were obtained for samples of more than 15 strains. We chose to phenotype and genotype 30 of the 50 strains that we isolated in total for each population (INRAE BIOGER collection), to ensure a sufficiently high statistical power and precision and to ensure that we could estimate allele frequencies and gene diversity in a population (Dale & Fortin, 2014).


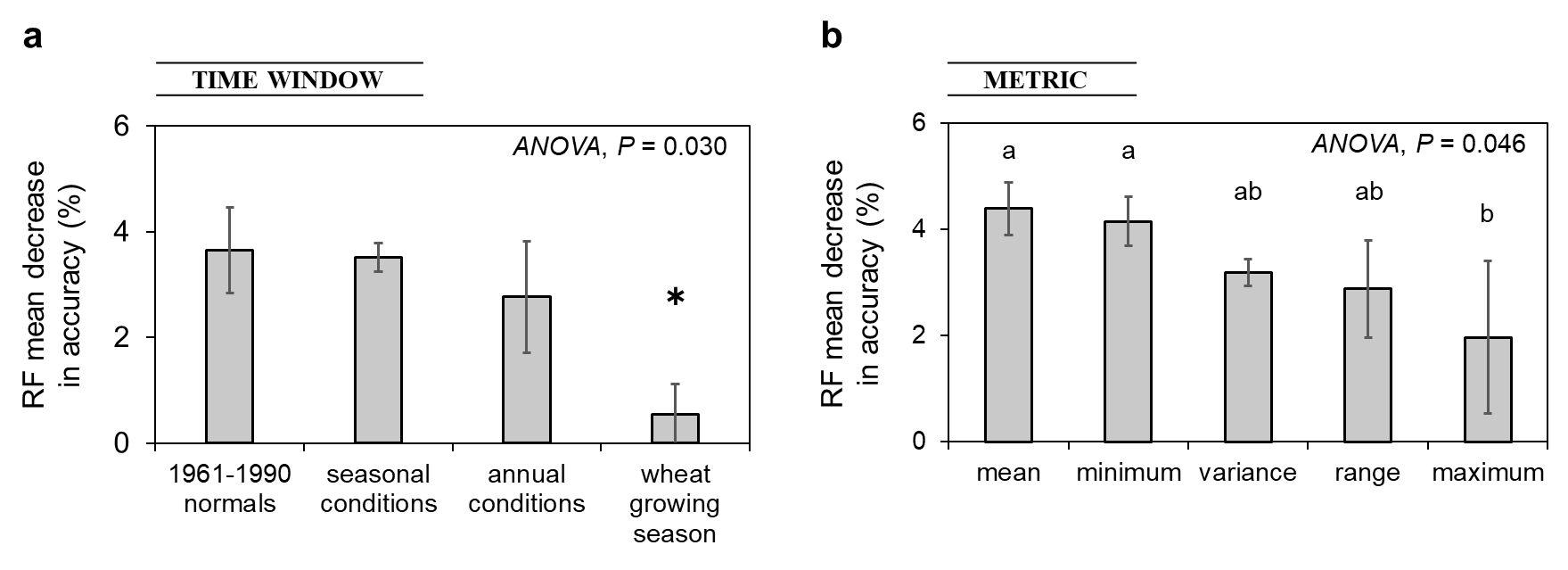


**Fig. S4 Characterisation of the thermal niche at each sampling site.** The mesoclimatic thermal environments of the 8 Euro-Mediterranean sampling locations were summarised (a) for four climatic time windows (‘1961-1990 climate normals’ and thermal conditions for the year of sampling averaged over the calendar year i.e. ‘annual conditions’, the ‘wheat growing season’ i.e. from late October to early July, the ‘seasonal conditions’ i.e. contrasts between the winter and spring periods) and (b) with five metrics (thermal mean, range, maximum, minimum and variance). We established a thermal niche (i.e. temperature conditions of a given sampling site) classification, by assessing the importance of each of the 20 thermal variables for discriminating between the three contrasting Köppen-Geiger climatic zones prospected, with a nonlinear and nonparametric random forest algorithm (RF; Breiman, 2001) in the ‘randomForest’ package of R (Liaw & Wiener, 2002). The importance of variables was compared on the basis of two metrics assessing the inaccuracy of RF zone classification if the variable concerned is not accounted for (RF mean decrease in prediction accuracy and node impurity, i.e. Gini coefficient). One-way analyses of variance (ANOVA outputs displayed with letters indicating the results of Tukey post-hoc multiple pairwise-comparisons) show that (a) the climatic time window of the wheat growing season and (b) maximum temperature are significantly less informative for classifying the three climatic zones. The difference between mean spring and mean winter temperatures gave the highest mean decrease in Gini index (0.27 *vs*. 0.25 for mean annual temperature over the 1961-1990 period), highlighting the importance of seasonal conditions in structuring the thermal responses of these geographic populations.


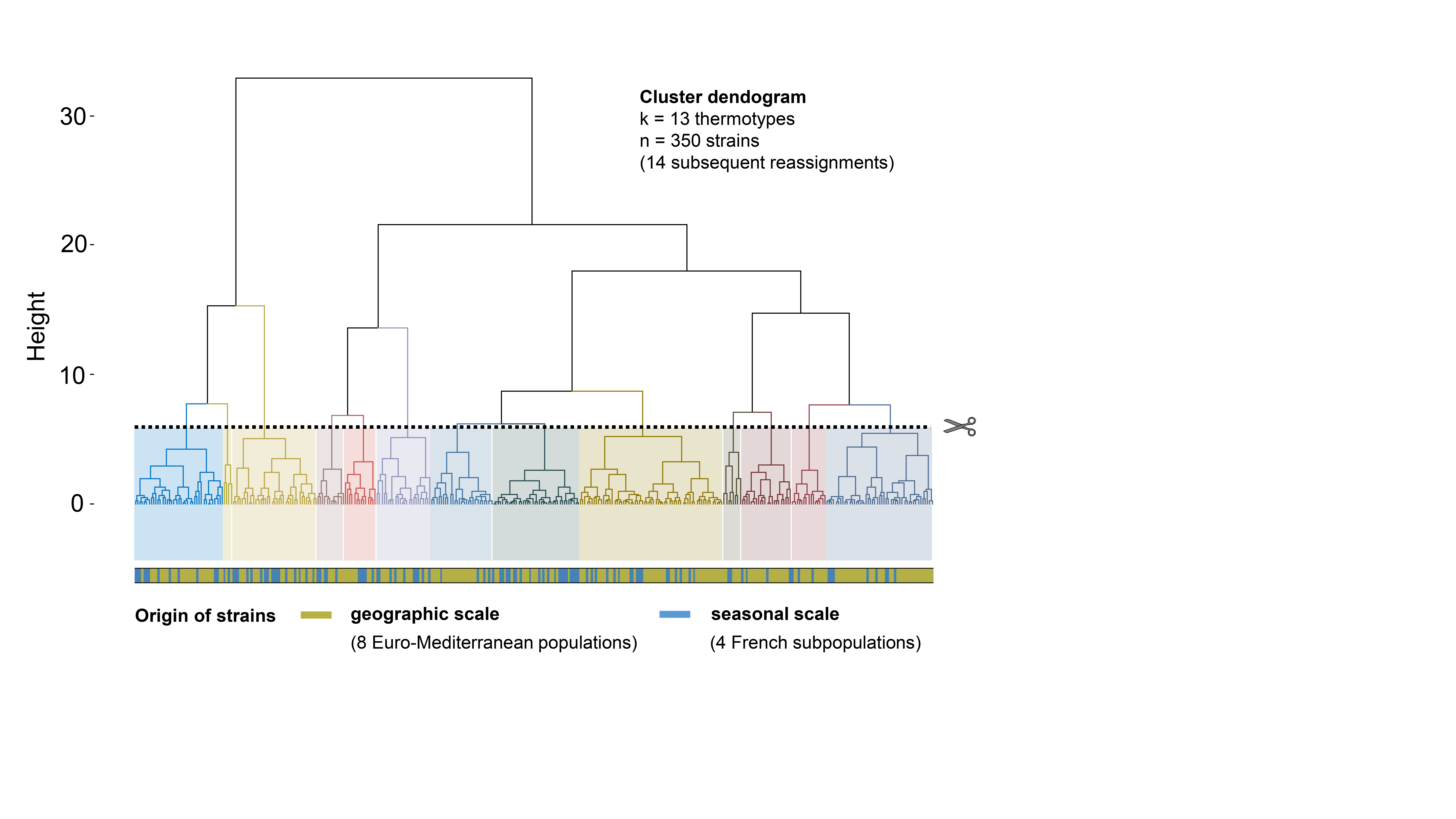


**Fig. S5 Clustering of *Z. tritici* strains into 13 thermotypes.** (a) The dendogram (distance-based tree) illustrates the hierarchical organisation of the TPCs of the 350 *Z. tritici* strains (seasonal and geographic scales) in terms of functional thermal responses. The distance between strains in the hierarchical cluster tree was calculated based on Euclidean distances (the greater the difference in height, the greater the dissimilarity). We identified 13 thermotypes, colour-coded in the figure, with the procedure presented in Methods S2. Each thermotype includes strains from both data sets (seasonal: blue segments; geographic: yellow segments in the horizontal bar below the tree). These results demonstrate the relevance of establishing a unique typology to compare the TPCs of all 350 strains. The quality of the clustering was assessed by silhouette analysis (Rousseeuw, 1987), based on the mean distance between clusters. 14 individuals were placed in the wrong cluster (negative silhouette width) and were therefore reassigned to the closest neighboring cluster before assessing the distribution and features (compactness, separation, connectivity) of each thermotype.


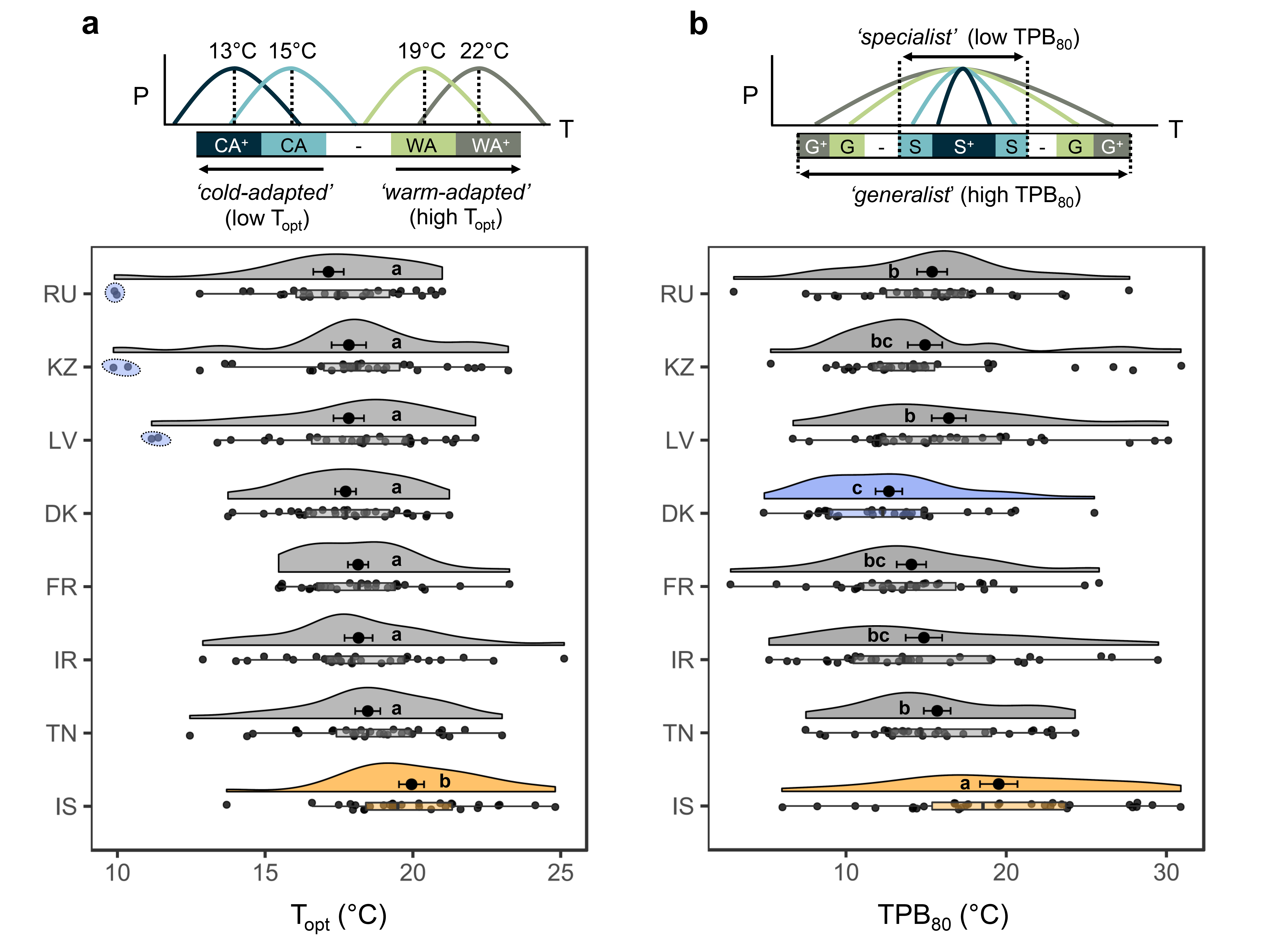


**
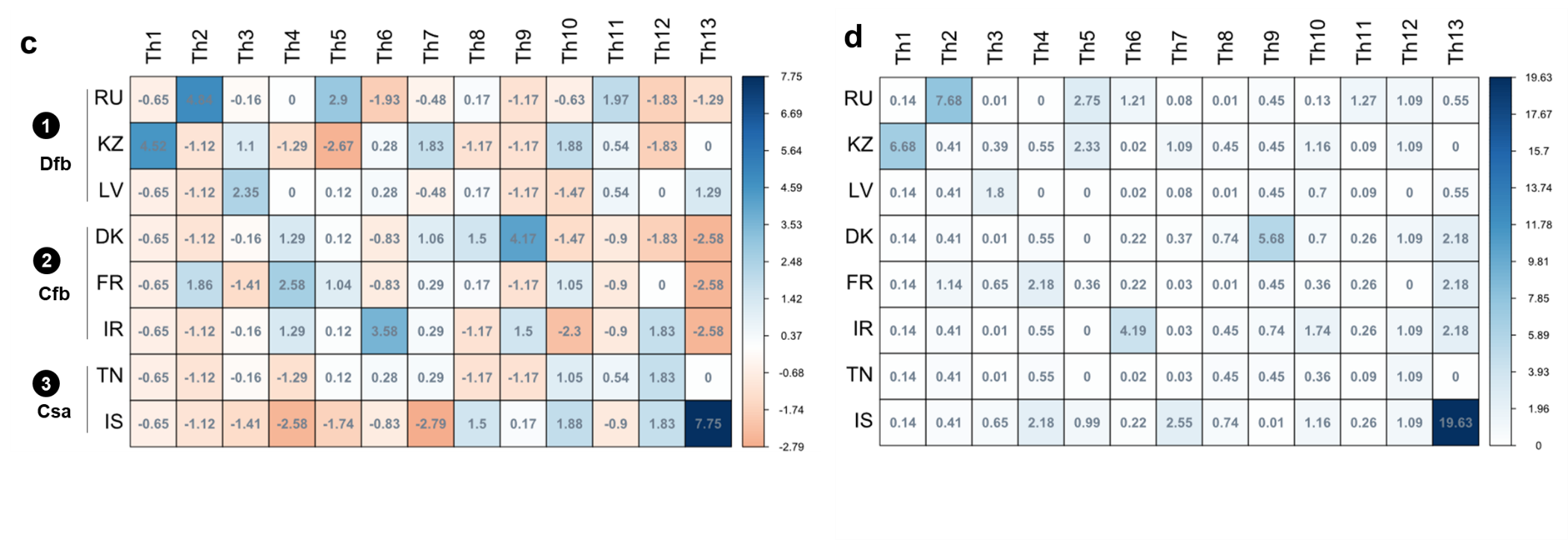
**

**Fig. S6 Distribution of thermotypes across the 8 Euro-Mediterranean *Z. tritici* populations** (sampled in RU: Russia; KZ: Kazakhstan; LV: Latvia; DK: Denmark; FR: France; IR: Ireland; TN: Tunisia; IS: Israel)**.** (a) Population-level thermal optima (means ± SEM) are presented, together with the distribution of individual T_opt_ values within populations (associated raw data points, boxplots and split-half violins). A significant shift in T_opt_ distribution towards higher temperature was detected for the Israeli population (IS); the letters indicate the outputs of Kruskal-Wallis post-hoc pairwise comparisons, with *P* < 0.05. (b) Population-level thermal breadth (means ± SEM) values are presented together with the variation in that response among individuals (associated raw data points, boxplots and split-half violins). The letters indicate the outputs of Kruskal-Wallis post-hoc pairwise comparisons, with *P* < 0.05. (c) Contingency table for the Chi-squared test of independence. Each row represents a population and each column represents a thermotype. Associations between rows and columns are displayed for each cell as Pearson residuals. Positive values in blue indicate an attraction and negative values in red indicate a repulsion. This table reveals that: (1) the most strongly “cold-adapted” thermotypes (CA^+^, Th1-Th2-Th3) are preferentially found in Dfb populations (RU-KZ-LV); (2) individuals with the greatest thermal breadth (G^+^, Th1 and Th13) are less abundant in Cfb populations (DK-FR-IR), which are characterised by more highly specialist individuals (S^+^, Th4 and Th9) than the average; (3) “warm-adapted” generalist individuals (WA^+^-G/G^+^, Th12,Th13) are found in higher proportions in the IS and, to a lesser extent, TN populations. (d) The relative contribution of each cell to the total Chi-squared score (darker cells are those corresponding to the bulk of the variability in thermotype distribution) indicates that the three aforementioned aspects account for (1) 17.7 %, (2) 17.1%, and (3) 21.8% of total phenotypic differentiation in thermal response between populations.


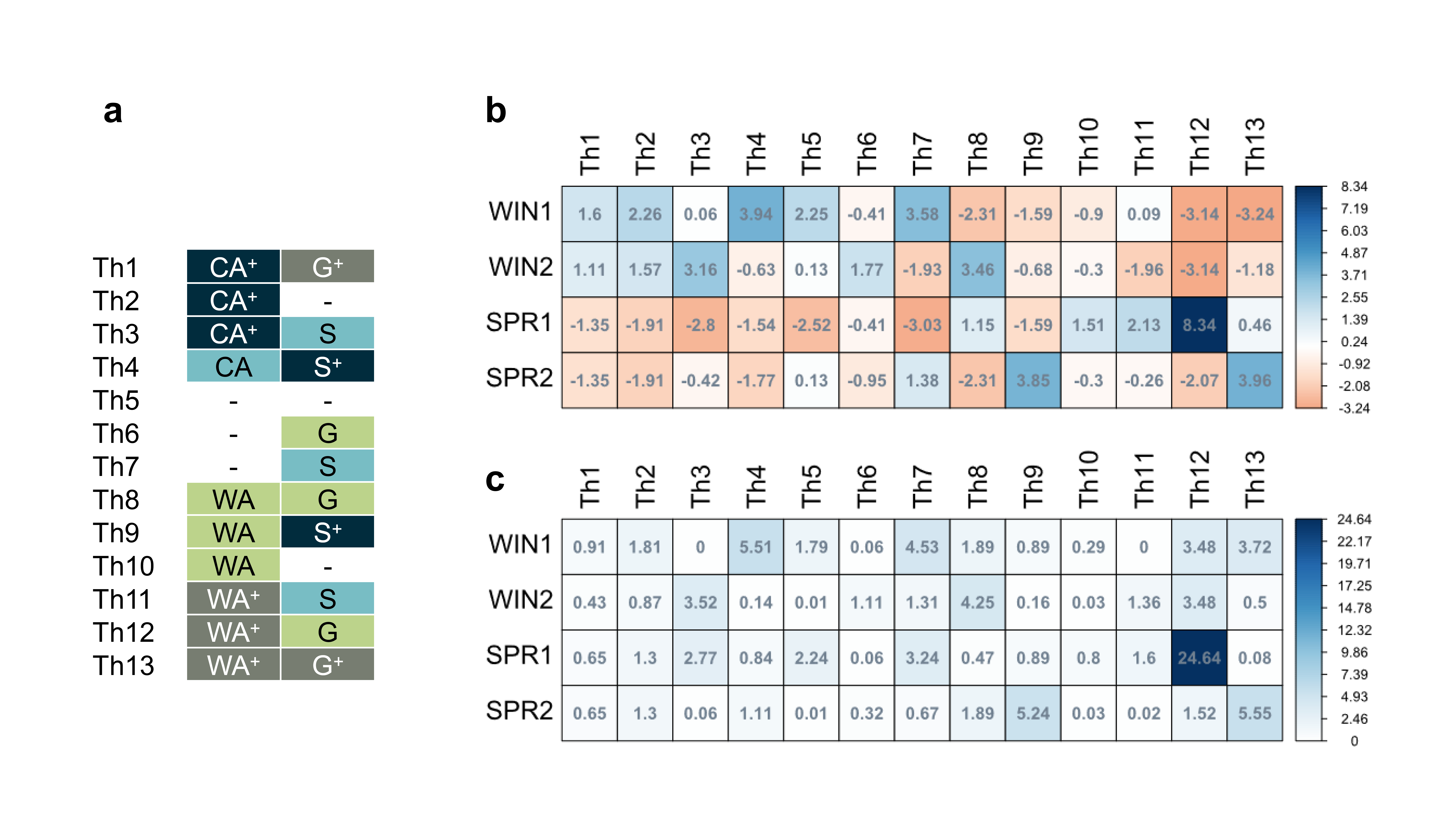


**Fig. S7** **Distribution of thermotypes across the four French seasonal *Z. tritici* subpopulations.** (a) Stacked bar plots of functional thermotype composition across populations: “highly cold-adapted” (CA^+^), “cold-adapted” (CA), “intermediate” (- in white), “warm-adapted” (WA), “highly warm-adapted” (WA^+^) thermotypes. The relative abundance within populations of each thermotype is expressed as a single bar percentage on which the thermotype ID is displayed. (b) Contingency table of the Chi-squared test of independence. Each row represents a population and each column represents a thermotype. Associations between rows and columns are displayed for each cell as Pearson residuals. Positive values in blue indicate an attraction and negative values in red indicate a repulsion. These residuals show that winter (WIN1 and WIN2) and spring (SPR1 and SPR2) subpopulations fall into discrete clusters in terms of their composition in thermotypes exhibiting higher performance under cold (CA^+^: positive association with winter populations and negative association with spring populations) and in thermotypes exhibiting higher performance under warm conditions (WA^+^: negative association with winter populations and positive association with spring populations). (c) The relative contribution of each cell to the total Chi-squared score (darker cells are those corresponding to the bulk of the variability in thermotype distribution) indicates that the compositional dissimilarity in thermotypes between winter and spring populations can be accounted for mostly by the proportions of generalist “warm-adapted” individuals (Th12 and Th13; 43% of the total Chi-squared score).

**
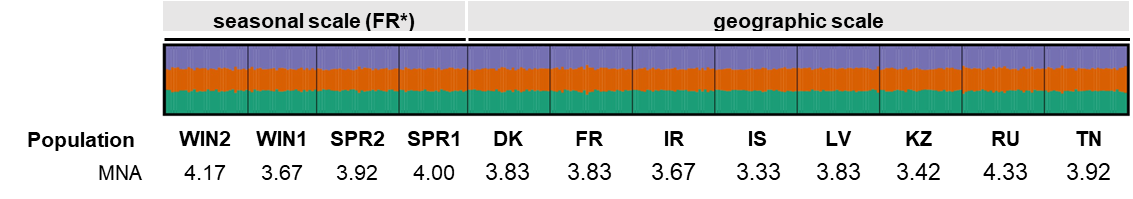
**

**Fig. S8** **Genetic diversity and population structure of the 12 *Z. tritici* populations.** The 350 *Z. tritici* strains were collected over a large spatial scale (8 geographic sites: DK: Denmark; FR: France; IR: Ireland; IS: Israel; KZ: Kazakhstan; LV: Latvia; RU: Russia; TN: Tunisia) and over a seasonal scale during another growing season in France (FR* with two pairs of post-winter (WIN1; WIN2) and post-spring (SPR1; SPR2) subpopulations). These strains were genotyped for 12 neutral microsatellite markers (SSRs) and assigned to different genetic clusters by a Bayesian cluster approach (see the detailed procedure in Methods S3). Each strain (one unique multilocus genotype) included in the analysis is displayed as a thin vertical line partitioned into colored segments representing its probabilities of assignment to genetically different clusters (cluster 1 in green, cluster 2 in orange and cluster 3 in purple). Each strain was affected to each of the three genetic structures with the same probability, indicating an absence of population structure. Genetic diversity is indicated for each population as the mean number of alleles observed over the 12 SSRs (MNA).


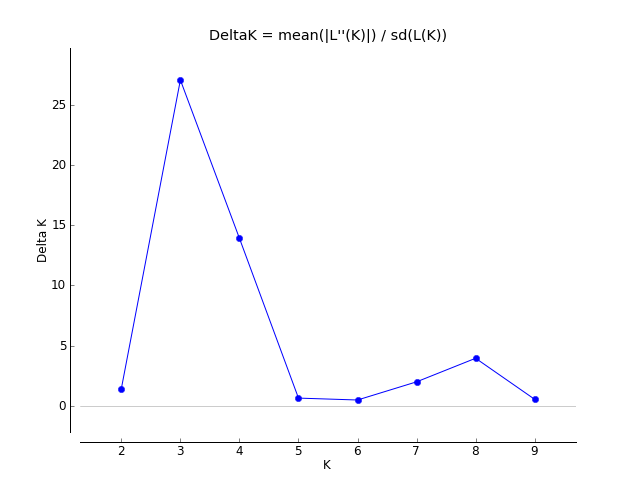


**Fig. S9 Delta K plot representing the most probable number of genetic groups within the full data set of 350 individuals.** The plot of mean likelihood of ΔK (rate of change in the log probability of data between successive K values) against the number of K groups was assessed to determine the number of genetic clusters that best fit the data based on the method described by Evanno *et al*. (2005). Here, ΔK calculations has a mode at K = 3.


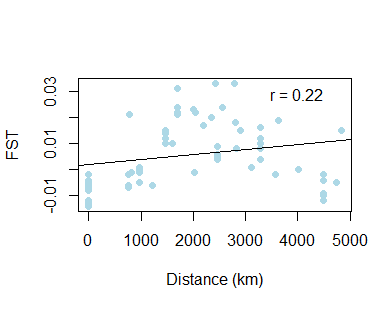


**Fig. S10 Correlation between genetic (F_ST_) and geographic distance among populations.** Although the correlation is non-significant (r = 0.22, *P* = 0.08), the least-squared linear regression line is shown for illustrative purposes.


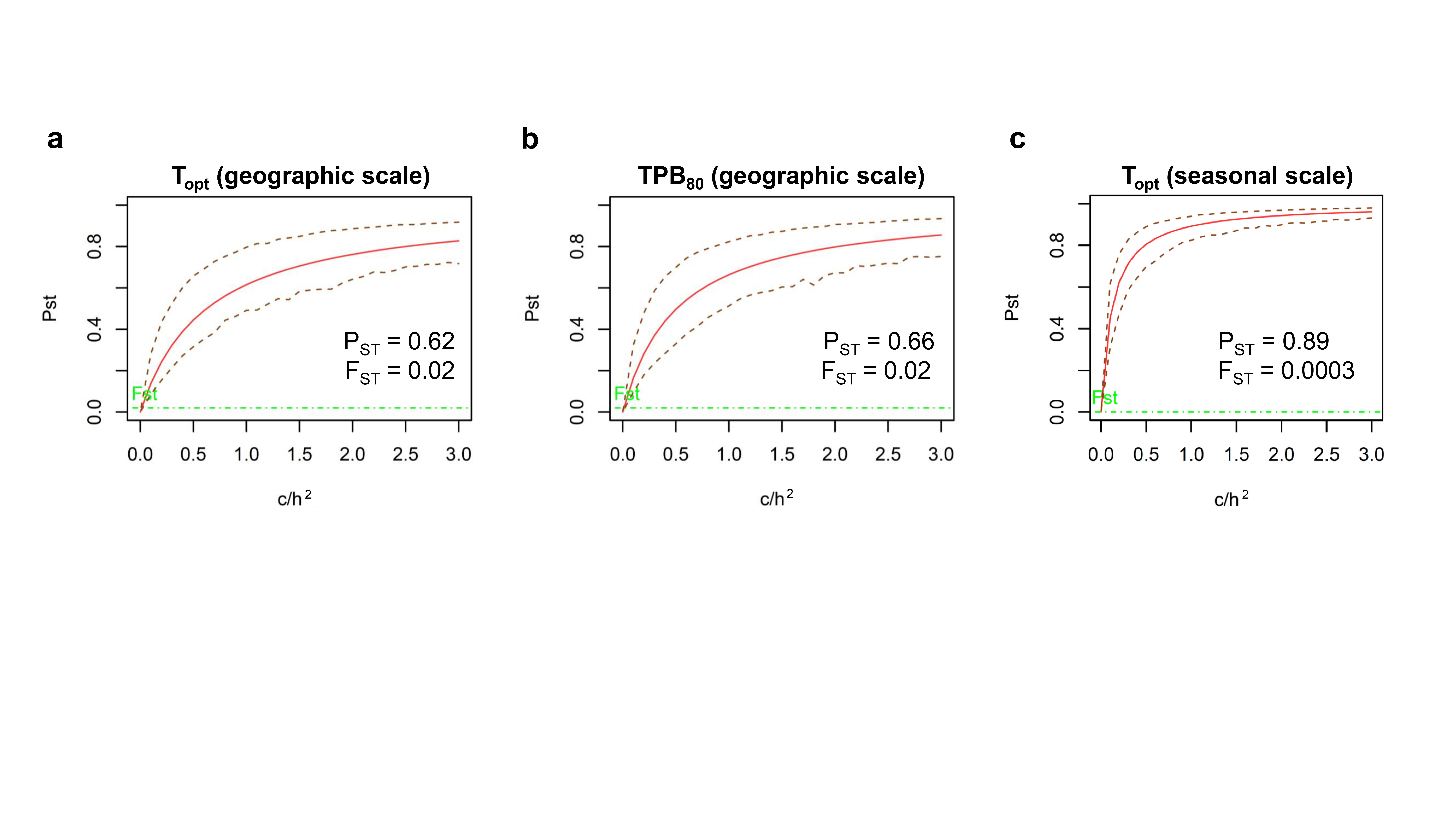


**Fig. S11 Sensitivity analysis of the robustness of P_ST_–F_ST_ comparisons for the 12 *Z. tritici* populations.** The robustness of P_ST_–F_ST_ comparisons was explored by studying variations of the c/h^2^ ratio, which determines the accuracy of the approximation of Q_ST_ by P_ST_. Phenotypic divergence in thermal sensitivity (P_ST_) was investigated for: (a) the thermal optimum (T_opt_) of the Euro-Mediterranean populations (geographic scale); (b) for the thermal breadth (TPB_80_) of Euro-Mediterranean populations; (c) for the thermal optimum (T_opt_) of the French seasonal subpopulations (seasonal scale). For each plot, estimates of P_ST_ (solid line in red, the P_ST_ value displayed being calculated at the critical c/h² ratio of 1), its lower and upper 95% confidence interval limits (dashed lines in red) and the upper confidence estimate of the neutral divergence (F_ST_; dashed line in green) are plotted. For each trait, the 95% CI of P_ST_ at c/h^2^ = 1 indicates the occurrence of strong phenotypic divergence and of a robust difference in P_ST_ and F_ST_, as their confidence intervals only overlap (P_ST_ > F_ST_) at low c/h^2^ ratios (a: c/h^2^ = 0.02; b: c/h^2^ = 0.02; c: c/h^2^ < 0.005).

**Table S1 Selection of a candidate mathematical model for establishing thermal performance curves (TPCs).** Model equations relating performance (P) to temperature (T) by means of K parameters were harmonised with the following key thermal parameters, when relevant: P_max_ (maximum performance), T_opt_ (thermal optimum), T_min_ (minimum temperature), T_max_ (maximum temperature), *Curv* (shape parameter) and TPB (thermal performance breadth). As in Boixel *et al.* (2019), the most appropriate model was selected to fit the TPC of each strain on the basis of: (i) statistic metrics accounting for goodness-of-fit namely residual sum of squares (RSS), Akaike weight (w_AIC_) and Schwarz weight (w_BIC_); (ii) the similarity between equation predictions of thermal responses over the temperature range and the best-fitting model (prediction dissimilarity: NS: not significant; ✱: *P* < 0.05) and their corresponding distribution of residuals along the temperature axis (with a view to maximising the accuracy of estimation for T_opt_ and the surrounding supra- and suboptimal estimates over the thermal extremes T_min_ and T_max_); (iii) model constraints potentially forcing the fitting process in a particular direction. Successive steps in the selection process are represented from the left to the right side of the table (black cells display discarded models). In this study of the thermal responses of *Z. tritici* over a Euro-Mediterranean spatio-temporal scale, as in the initial study performed in France, no significant difference in fit quality was observed between the quadratic and beta models for growth rate data over the entire range of temperatures investigated (from 6.5 to 33.5°C). However, their residuals were not similarly distributed along the temperature-axis, leading to small differences between equations in estimation accuracy for performance at mid-temperature ranges relative to outer edges (see the column ‘distribution of residuals’ below and in ESM2 of Boixel *et al.*, 2019). In both studies, we selected the model that best estimated performance over the mid-temperature range (here the quadratic model), as diversity was assessed on three thermal traits extracted from this range of the TPC (P_max_, T_opt_, TPB_80_).

| **Mathematical models** | **Relationship between**  **temperature (T) and performance (P)** | | **K** | **Goodness of fit over the temperature range** | | | | | **Model constraints** | | **Final**  **choice** | |
| --- | --- | --- | --- | --- | --- | --- | --- | --- | --- | --- | --- | --- |
|  |  |  |  | **RSS** | **w_AICc_** | **w_BIC_** | **Prediction**  **dissimilarity** | **Distribution**  **of residuals** |  |  |  |  |
| **Kontodimas**  (modified Analytis equation) | $P\left( T \right)=a\times(T-T_{min})^{2}\times(T_{max}-T)$ | | 3 | 119.3 | 0.24 | 0.31 | NS | Mid-range |  | |  | |
| **Quadratic** | $P\left( T \right)=P_{max}+Curv(T-T_{opt})^{2}$ | | 3 | 119.9 | 0.21 | 0.26 | NS | Outer edge  (inner boundary) | Symmetry | | ✓ | |
| **Yan and Hunt**  (modified beta distribution) | $P\left( T \right)=P_{max}(\frac{T_{max}-T}{T_{max}-T_{opt}})(\frac{T}{T_{opt}})^{T_{opt}/(T_{max}-T_{opt})}$ | | 3 | 119.7 | 0.22 | 0.27 | NS | Outer edge  (outer boundary) | T_min_=0 | |  | |
| **Baker *et al.*** | $P\left( T \right)=ae^{bT}(T_{max}-T)(T-T_{min})$ | | 4 | 119.5 | 0.07 | 0.03 | ✱ |  |  | |  | |
| **Beta distribution** | $P\left( T \right)=P_{max}((\frac{T_{max}-T}{T_{max}-T_{opt}})(\frac{T-T_{min}}{T_{opt}-T_{min}})^{\frac{\left( T_{opt}{-T}_{min} \right)}{\left( T_{max}-T_{opt} \right)}})$ | | 4 | 119.7 | 0.07 | 0.03 | NS | Mid-range upper limits |  | |  | |
| **Modified Gaussian** | $P\left( T \right)=P_{max}e^{(-0.5(\frac{\left\vert T-T_{opt} \right\vert}{TPB})^{d}}$ | 4 | | 118.3 | 0.10 | 0.05 | ✱ |  | |  | |  |
| **Square-root model**  (Ratkowsky equation) | $P\left( T \right)=(a(T-T_{min})(1-e^{b\left( T-T_{max} \right)}))^{2}$ | 4 | | 134.4 | 0.01 | 0.01 | ✱ |  | |  | |  |
| **Third-order polynomial** | $P\left( T \right)=a+b{T+cT^{2}+dT}^{3}$ | 4 | | 119.3 | 0.08 | 0.04 | ✱ |  | |  | |  |

**Table S2 Population-pairwise genetic distance (matrix of F_ST_ values).** Pairwise comparisons between the 8 geographic *Z. tritici* populations (DK: Denmark; FR: France; IR: Ireland; IS: Israel; KZ: Kazakhstan; LV: Latvia; RU: Russia; TN: Tunisia) and 4 seasonal *Z. tritici* subpopulations (WIN1, WIN2: post-winter; SPR1, SPR2: post-spring). The matrix displays the estimation of pairwise F_ST_-values for each combination of populations based on 12 neutral microsatellite markers. Values in italics indicate significant F_ST_ values (*P* < 0.05), as evaluated with random allelic permutation procedures (1,023 permutations). These low population pairwise F_ST_-values and associated statistical analyses (conducted with the ARLEQUIN program; see Methods S3) indicate an absence of genetic differentiation between populations based on neutral markers (exact global test of sample differentiation based on haplotype frequencies conducted with 100,000 Markov steps: *P* = 0.75 ± 0.11)

|  | WIN2 | WIN1 | SPR2 | SPR1 | DK | FR | IR | IS | LV | KZ | RU | TN |
| --- | --- | --- | --- | --- | --- | --- | --- | --- | --- | --- | --- | --- |
| WIN2 | 0 |  |  |  |  |  |  |  |  |  |  |  |
| WIN1 | -0.013 | 0 |  |  |  |  |  |  |  |  |  |  |
| SPR2 | -0.007 | -0.008 | 0 |  |  |  |  |  |  |  |  |  |
| SPR1 | -0.012 | -0.014 | *-0.002* | 0 |  |  |  |  |  |  |  |  |
| DK | *-0.005* | *-0.005* | *0.001* | *0.000* | 0 |  |  |  |  |  |  |  |
| FR | -0.006 | *-0.004* | *-0.004* | *-0.005* | *-0.001* | 0 |  |  |  |  |  |  |
| IR | -0.007 | -0.006 | *-0.006* | *-0.002* | -0.006 | -0.006 | 0 |  |  |  |  |  |
| IS | *0.012* | *0.010* | *0.008* | *0.016* | *0.001* | *0.004* | *-0.000* | 0 |  |  |  |  |
| LV | *0.024* | *0.022* | *0.031* | *0.021* | *0.021* | *0.021* | *0.023* | *0.033* | 0 |  |  |  |
| KZ | -0.012 | -0.010 | *-0.002* | -0.009 | *-0.002* | *-0.004* | *-0.005* | *0.019* | *0.018* | 0 |  |  |
| RU | *0.004* | *0.006* | *0.009* | *0.005* | *0.010* | *0.006* | *0.008* | *0.024* | *-0.001* | *-0.001* | 0 |  |
| TN | *0.014* | *0.014* | *0.015* | *0.010* | *0.022* | *0.012* | *0.017* | *0.020* | *0.033* | *0.015* | *0.015* | 0 |

**Table S3 Hierarchical analysis of molecular variance results (AMOVA)** partitioning genetic variation within and among populations, performed with ARLEQUIN v3.5 (Excoffier and Lischer, 2010).

| Source of variation | Sum of squares | Variance components | Percentage of variation |
| --- | --- | --- | --- |
| Among populations | 57.3 | 0.02 | 0.61 |
| Within populations | 2644.7 | 3.84 | 99.39 |
| Total | 2702.0 | 3.87 |  |

**
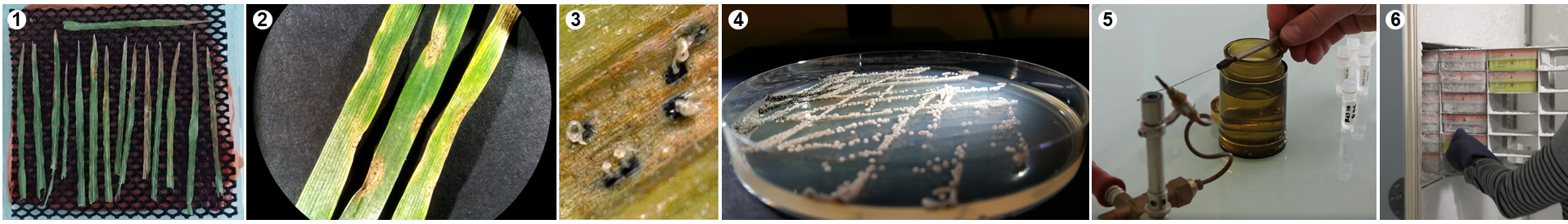
**

**Methods S1** **Procedure for the sampling, collection and recovery of *Zymoseptoria tritici* strains.** Leaves with Septoria tritici blotch lesions were sampled at the various field locations, dried and flattened naturally for 2-3 days between two pieces of blotting paper, (1) placed overnight at 18 °C in a humidity chamber to promote the extrusion of cirrhi and then (2) observed with a binocular magnifier to visualise pycnidia. (3) On each leaf, one cirrhus from a single pycnidium was retrieved from a randomly sampled single lesion, for further isolation in pure culture through (4) subculturing a single colony on PDA (potato dextrose agar, 39 g L^−1^) at 18°C in the dark. (5) After purification, *Z. tritici* spore suspensions were immediately put in stock tubes, in a 1:1 glycerol–water mixture, and were added to (6) the INRAE BIOGER *Z. tritici* collection, which is stored at -80 °C.


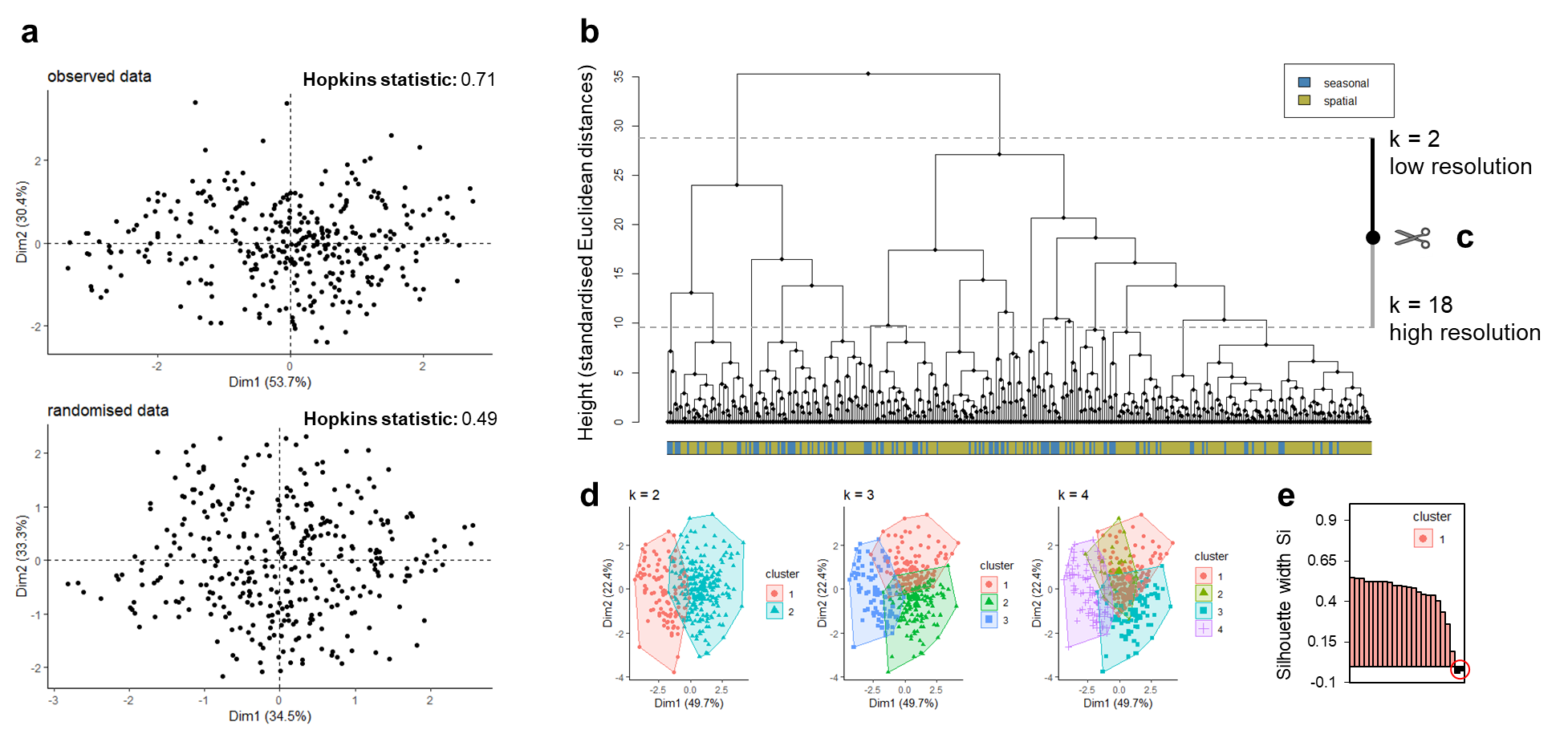


**Methods S2 Definition of *Z. tritici* ‘thermotypes’.** Thermotypes should be considered here as functional groups of thermal performance curves (TPCs) with similar thermal sensitivity features. Thermotype definition involves the establishment of a typology accounting for the diversity encountered in a given data set. This typology was built on the standardised phenotyping multidimensional data (including P_max_, T_opt_ and TPB_80_ for both seasonal and geographic populations) in a five-step approach: (a) testing for uniformity in the data by calculating the Hopkins statistic (which measures spatial randomness and, thus, the tendency of a given data set to cluster; Lawson & Jurs, 1990) to determine whether a given data set can be divided into meaningful clusters; (b) calculating Euclidean distances between pairs of individual phenotypes and building the corresponding distance-based tree; (c) determining the optimal number of clusters (the cut-off being represented by the pair of scissors) by varying k from k = 2 (as we highlighted that there are clusters in the data set; see the Hopkins statistic in S2a) to k = 18 (to account for all combinations of variables: P_max_ × T_opt_ × TPB_80_). We achieved this by data mining, by identifying the best clustering scheme using 30 clustering validity indices implemented in the ‘*NbClust*’ R package (Charrad *et al.*, 2014); (d) K-means clustering, an iterative algorithm with the objective function of minimising the pooled mean distances within clusters. This approach identified the 13 clusters to be generated into which the data could be partitioned (‘*kmeans*’ function with 13 centers and 50 initial configurations); (e) assessing the quality of the clustering result, by determining how well observations clustered with the ‘*silhouette*’ function of the ‘*cluster*’ package of R (Maechler *et al.*, 2018). Based on this silhouette analysis (Rousseeuw, 1987), outliers among the thermal responses clustered (i.e. wrongly assigned phenotypes with a negative silhouette width coefficient – circled in red in the figure) were reassigned to the neighbouring cluster. This final partitioning of data (clustering result with reassigned outliers) was used to compare the corresponding clusters (*i.e.* thermotypes) on the basis of their features (compactness, separation, connectivity within and between clusters), abundance and distribution pattern within and between the 12 *Z. tritici* populations.


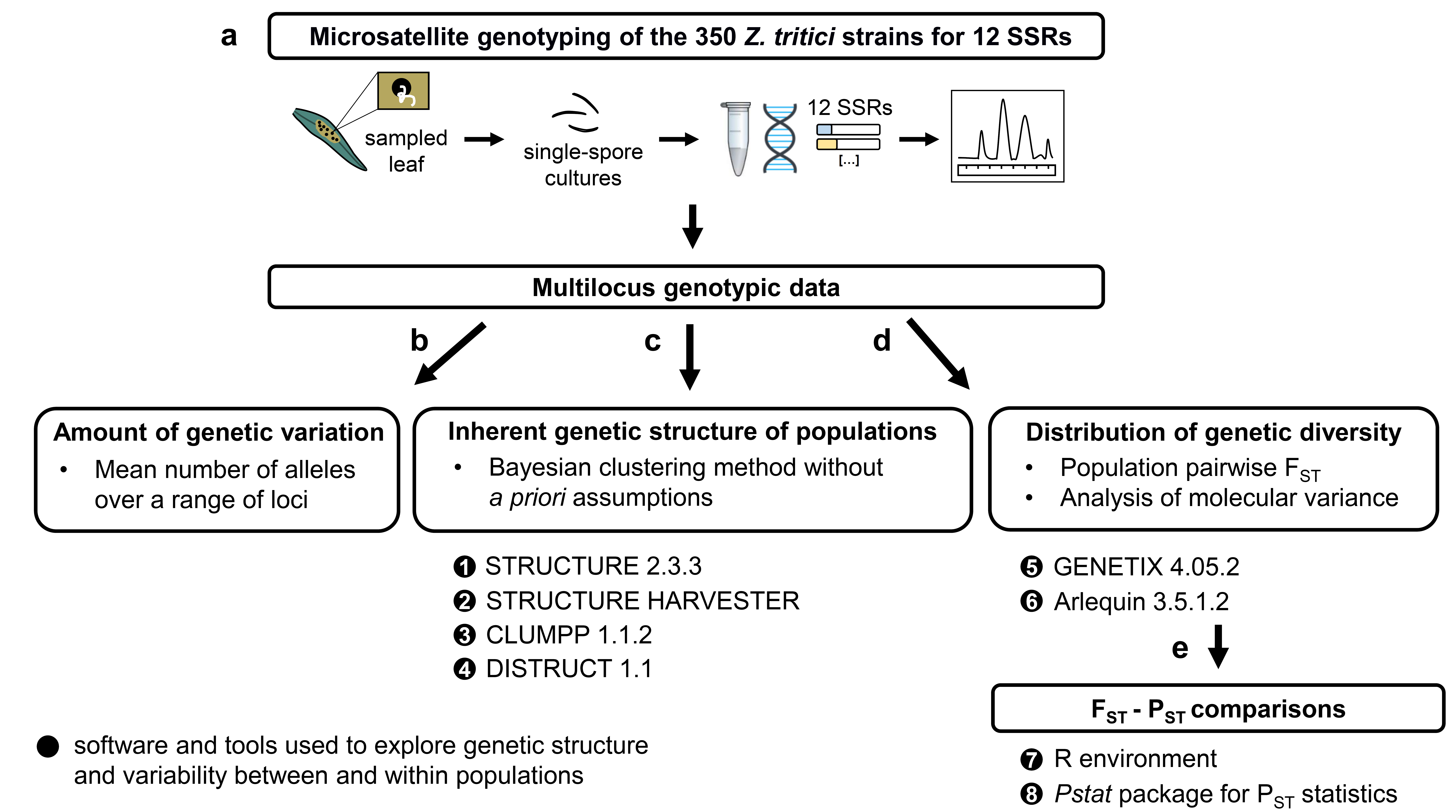


**Methods S3 Procedure for acquiring and analysing multilocus genotypic data.** (a) Genetic variability, structure and the distribution of diversity between and within the 12 *Z. tritici* populations were assessed based on microsatellite genotyping data. The overall data set was acquired for 12 SSRs (ST1, ST2, ST3A, ST3B, ST3C, ST4, ST5, ST6, ST7, ST9, ST10, ST12 neutral microsatellites; Gautier *et al.*, 2014) following: (i) DNA extraction from single-spore cultures with the DNeasy Plant Maxi Kit (QIAGEN); (ii) amplification and sequencing of the SSR markers in one multiplex PCR sample (Eurofins Analytics France); (iii) the determination and annotation of allele sizes through visual analysis of individual chromatograms with Peak Scanner (Applied Biosystems). (b) Genetic variation in allelic distributions was assessed manually by the direct counting of the mean number of alleles (MNA) observed over all loci. (c) The distribution of this genetic variation within and between populations (population structure) was inferred with the Bayesian clustering approach implemented in STRUCTURE (Pritchard *et al.*, 2000) under the admixture and correlated allele frequencies model. The algorithm was run on the basis of 500,000 iterations of the Markov chain (‘burn-in length’) followed by a run phase of 1,000,000 iterations (‘burn-in period’) with 10 independent replicates for each tested number of clusters (range set from 1 to 10). The optimal number of inferred genetic clusters (K) was estimated by the Evanno method (Evanno *et al.*, 2005) with STRUCTURE HARVESTER (Earl & vonHoldt, 2012). Output data were then visualised with CLUMPP (Jakobsson & Rosenberg, 2007) and DISTRUCT (Rosenberg, 2003). (d) The genetic divergence between the sampled populations (genetic differentiation assessed on pairwise estimates of Weir and Cockerham's F-statistic: 1,000 randomisations) was calculated with GENETIX (Belkhir, 2004). Hierarchical analyses of molecular variance (AMOVA) were conducted with Arlequin (Excoffier & Lischer, 2010) to assess the contribution of sampling date and location to the differences and patterns of genetic variance detected. (e) For inferences of the contribution of genetic drift and natural selection to the variation in thermal responses between populations, P_ST_ values (phenotypic differentiation between populations) of P_max_ (maximum performance), T_opt_ (thermal optimum), TPB_80_ (thermal performance breadth) and their confidence intervals were calculated with the ‘*Pstat’* package of R (using the ‘*TracePst’* function under the arguments boot = 1000 and pe = 0.95; Da Silva & Da Silva, 2018). F_ST_-P_ST_ comparisons were performed to test for patterns of local adaptation and to assess whether phenotypic differentiation between populations were greater or smaller than expected under the influence of genetic drift.

**ADDITIONAL REFERENCES**

**Belkhir K**. **2004**. *GENETIX 4.05, logiciel sous Windows^TM^ pour la génétique des populations.*

**Boixel A-L, Delestre G, Legeay J, Chelle M, Suffert F**. **2018**. Phenotyping thermal responses of yeasts and yeast-like microorganisms at the individual and population levels: proof-of-concept, development and application of an experimental framework to a plant pathogen. *Microbial Ecology*.

**Breiman L**. **2001**. Random forests. *Machine Learning* **45**: 5–32.

**Charrad M, Ghazzali N, Boiteau V, Niknafs A**. **2014**. NbClust: An R Package for Determining the Relevant Number of Clusters in a Data Set. *Journal of Statistical Software* **61**: 1–36.

**Da Silva SB, Da Silva A**. **2018**. Pstat: an R package to assess population differentiation in phenotypic traits. *The R Journal* **10**: 447–454.

**Dale MRT, Fortin M-J**. **2014**. *Spatial analysis: a guide for ecologists*. Cambridge, UK: Cambridge University Press.

**Earl DA, vonHoldt BM**. **2012**. STRUCTURE HARVESTER: a website and program for visualizing STRUCTURE output and implementing the Evanno method. *Conservation Genetics Resources* **4**: 359–361.

**Evanno G, Regnaut S, Goudet J**. **2005**. Detecting the number of clusters of individuals using the software structure: a simulation study. *Molecular Ecology* **14**: 2611–2620.

**Excoffier L, Lischer HEL**. **2010**. Arlequin suite ver 3.5: a new series of programs to perform population genetics analyses under Linux and Windows. *Molecular Ecology Resources* **10**: 564–567.

**Gautier A, Marcel TC, Confais J, Crane C, Kema G, Suffert F, Walker A-S**. **2014**. Development of a rapid multiplex SSR genotyping method to study populations of the fungal plant pathogen *Zymoseptoria tritici*. *BMC Research Notes* **7**: 373.

**INRAE AgroClim**. **2019**. Monitoring data from the INRAE CLIMATIK platform. *https://intranet.inrae.fr/climatik/*.

**Jakobsson M, Rosenberg NA**. **2007**. CLUMPP: a cluster matching and permutation program for dealing with label switching and multimodality in analysis of population structure. *Bioinformatics* **23**: 1801–1806.

**Köppen W**. **1936**. Das geographische system der klimat. In: Handbuch der klimatologie. 46.

**Lawson RG, Jurs PC**. **1990**. New index for clustering tendency and its application to chemical problems. *Journal of chemical information and computer sciences* **30**: 36–41.

**Liaw A, Wiener M**. **2002**. Classification and regression by randomForest. *R news* **2**: 18–22.

**Maechler M, Rousseeuw P, Struyf A, Hubert M, Hornik K**. **2018**. *cluster: Cluster Analysis Basics and Extensions*.

**Norwegian Meteorological Institute**. **2019**. Online weather service from the Norwegian Meteorological Institute and the Norwegian Broadcasting Corporation. *http://www.yr.no/*.

**Peel MC, Finlayson BL, McMahon TA**. **2007**. Updated world map of the Köppen-Geiger climate classification. *Hydrology and Earth System Sciences Discussions* **4**: 439–473.

**Pritchard JK, Stephens M, Donnelly P**. **2000**. Inference of population structure using multilocus genotype data. *Genetics* **155**: 945.

**Rosenberg NA**. **2003**. DISTRUCT: a program for the graphical display of population structure. *Molecular Ecology Notes* **4**: 137–138.

**Rousseeuw PJ**. **1987**. Silhouettes: a graphical aid to the interpretation and validation of cluster analysis. *Journal of Computational and Applied Mathematics* **20**: 53–65.

**Suffert F, Goyeau H, Sache I, Carpentier F, Gélisse S, Morais D, Delestre G**. **2018**. Epidemiological trade-off between intra- and interannual scales in the evolution of aggressiveness in a local plant pathogen population. *Evolutionary Applications* **11**: 768–780.

**Zhan J, McDonald BA**. **2011**. Thermal adaptation in the fungal pathogen *Mycosphaerella graminicola*. *Molecular Ecology* **20**: 1689–1701.
